# Long-term warming raises risks of seasonal seafloor methane release in the coastal Baltic Sea

**DOI:** 10.3389/fmicb.2025.1636301

**Published:** 2025-10-07

**Authors:** Songjun Li, Marcelo Ketzer, Cheng Chang, Iryna Rula, Laura Seidel, Ida Krogsgaard Svendsen, Anders Forsman, Samuel Hylander, Mark Dopson

**Affiliations:** ^1^Centre for Ecology and Evolution in Microbial Model Systems (EEMiS), Linnaeus University, Kalmar, Sweden; ^2^Department of Biology and Environmental Sciences, Linnaeus University, Kalmar, Sweden; ^3^Department of Ecology, Environment and Plant Sciences, Stockholm University, Stockholm, Sweden

**Keywords:** climate change, methane, sulfate, sediment, 16S rRNA gene

## Abstract

Climate change driven ocean warming is a worldwide environmental issue that can impact cycling of greenhouse gases. However, how methane production in marine sediments as a potential contributor to atmospheric greenhouse gases versus its consumption at the sulfate–methane transition zone will be affected by climate change related warming is still not well constrained. In this study, sediments from two Baltic Sea bays with long-term temperature differences were collected during summer and winter. The primary difference between the two bays was that one had been heated by a nearby power plant for 50 years, resulting in a 5.1 °C increase in annual average temperature compared to an unheated control bay. The results showed that near-seafloor sediment methane concentrations were 50 times higher compared to present-day conditions. Furthermore, the sediment fluxes along with microbial community composition changes suggested that long-term warming may thin the sulfate reduction zone, such that methanotrophic archaea and sulfate reducing bacteria peaked at shallower sediment depths in the heated bay. Overall, the results from long-term warming in natural sediment environment indicated that future climate change warming may increase the risk of methane release to the water and eventually the atmosphere.

## 1 Introduction

In the last century, climate change has become a serious environmental problem that has triggered a cascade of consequences in the marine system such as rising ocean temperature (Doney et al., 2012), deoxygenation (Breitburg et al., 2018), ocean acidification (Orr et al., 2005), and sea level rise (DeConto and Pollard, 2016). Despite comprising only ~3% of the total marine area, coastal zones provide nearly half the global oceanic primary production (Paerl, 1997). Furthermore, these ecosystems provide other important services such as carbon sequestration and storage (Martínez et al., 2007), fisheries production, and habitats of high biodiversity (Luisetti et al., 2014). The Baltic Sea is a brackish inland water body with limited connection to the open sea (Kullenberg and Jacobsen, 1981). Baltic Sea surface temperatures have increased by 0.6 °C per decade between 1990–2008 and are predicted to increase between 1.9 and 3.2 °C by the end of 21st century (Meier, 2006). In addition, the dissolved oxygen level has decreased due to eutrophication and increased temperature, such that hypoxic/anoxic areas or “dead zones” have expanded 10-fold within the last century (Carstensen et al., 2014).

Methane (CH_4_) is the second most important greenhouse gas after carbon dioxide (CO_2_) and has a greater than 20-fold warming potential compared to CO_2_ (Saunois et al., 2019). Methane from marine sediments is a rising source in the atmospheric budget under climate change, with shallow coastal areas contributing the majority of emissions (Borges et al., 2016; Weber et al., 2019). Processes generating methane in coastal settings include microbial methanogenesis in sediment (Bange et al., 1994; Rehder et al., 1998) that can be transferred to the water and to the atmosphere via gas bubbles or diffusion through the seafloor (Hermans et al., 2024). Higher marine productivity related to climate change warming is predicted to intensify the incidence of dead zones (Altieri and Gedan, 2015) as well as shallowing geochemical zones such that methane generation occurs closer to the sediment surface (Seidel et al., 2023b). While methane emissions are known to be high after summer heatwaves (Humborg et al., 2019), it is unknown how year-round methane fluxes will alter in the face of climate change related long-term warming.

Methane is consumed via microbial oxidation to sustain their growth in a process termed “methanotrophy” (Hanson and Hanson, 1996). In anoxic sediment environments, anaerobic oxidation of methane (AOM) couples methane oxidation to, e.g., sulfate reduction (Knittel and Boetius, 2009) to consume most global seafloor sedimentary methane (i.e., 45–61 Tg CH_4_) in water depths <200 m (Egger et al., 2018). AOM occurs at the sediment sulfate–methane transition zone, termed “SMTZ” (Knittel and Boetius, 2009), which is the transition between the overlying microbial sulfate reduction and underlying methane production (Berner, 1981; Niewöhner et al., 1998; Jørgensen et al., 2001). AOM often occurs as a syntrophic process between two microbial groups, an anaerobic methanotrophic (ANME) archaea and a sulfate reducing bacteria (SRB) whereby electrons are directly passed from the ANME to the SRB (Hinrichs et al., 1999; Orphan et al., 2001; Knittel and Boetius, 2009). Methane and sulfate fluxes in the Baltic Sea estuarine sediments have seasonal variability, with high values in summer and lower in winter (Sawicka and Brüchert, 2017) that are controlled by factors such as the organic matter deposition on the sea-floor and temperature (Ketzer et al., 2024). However, how methanogenesis/sulfate reduction along with the microbial communities responsible for AOM in the SMTZ might alter in the future under a warming climate remains largely unknown.

The underlying study was conducted in a Baltic Sea bay that has been heated for the past 50 years by receiving warm water from the cooling system of a nearby power plant. The plant discharges warmed water causing an average increased water temperature in relation to an adjacent control bay of 5.1 °C (Seidel et al., 2022). Aside from the water temperature and related biogeochemical parameters, the characteristics of the two bays are essentially similar due to their close geographical proximity (Seidel et al., 2022). This makes the heated and control bays an ideal system to investigate the influence of long-term, climate-related warming in a large-scale Baltic Sea coastal area undergoing naturally seasonal fluctuations. Previous research in this system found that long-term warming alters the diversity and vertical structure of the sediment microbial communities and increases the downward flux of sulfate from the seafloor into the sediments in the heated bay (Seidel et al., 2023b). Sulfate fluxes showing a linear profile with decreasing concentrations with depth are indicative of AOM-dominated sulfate reduction and therefore, are also used as a proxy for an equivalent upward methane flux (Ketzer et al., 2024). Moreover, the increased diversity and relative abundance of methanogens in shallower depths in heated bay sediment corroborates augmented sulfate fluxes and a shallowing of the SMTZ (Seidel et al., 2023a). In turn, this could lead to higher methane concentrations produced by the microbial community at shallower depths, increasing the potential for seafloor methane release. Other studies in Brazil have found similar patterns, with methane accumulating at shallow sediment depths (Benites et al., 2015), and the diversity of sediment microbial communities influenced by sediment depth, methane concentrations, and organic matter concentrations (Romano et al., 2021). However, how the SMTZ will shift along with inter-related influences on methane fluxes to the water column has not been established.

The aims of this study were to investigate sediment methane and sulfate concentrations and fluxes along with their related microbial community’s response to long-term warming in summer and winter in a natural system with annual variations that closely reflect future warming scenarios. The selection of summer and winter for this study was due to summer representing extremely high temperatures, often exceeding 30 °C, while winter was the season characterized by the greatest temperature variation between the two bays. It was hypothesized that: (1) the sediment methane and sulfate fluxes have variability between summer and winter in both the heated and control bay; (2) the difference in methane and sulfate fluxes between the two bays was less pronounced in summer compared to winter as the temperature difference was less in summer; and (3) the microbial communities differed due to the variation in methane and sulfate fluxes between the bays in summer and winter.

## 2 Materials and methods

### 2.1 Sampling sites and sediment slicing

**Figure 1:**
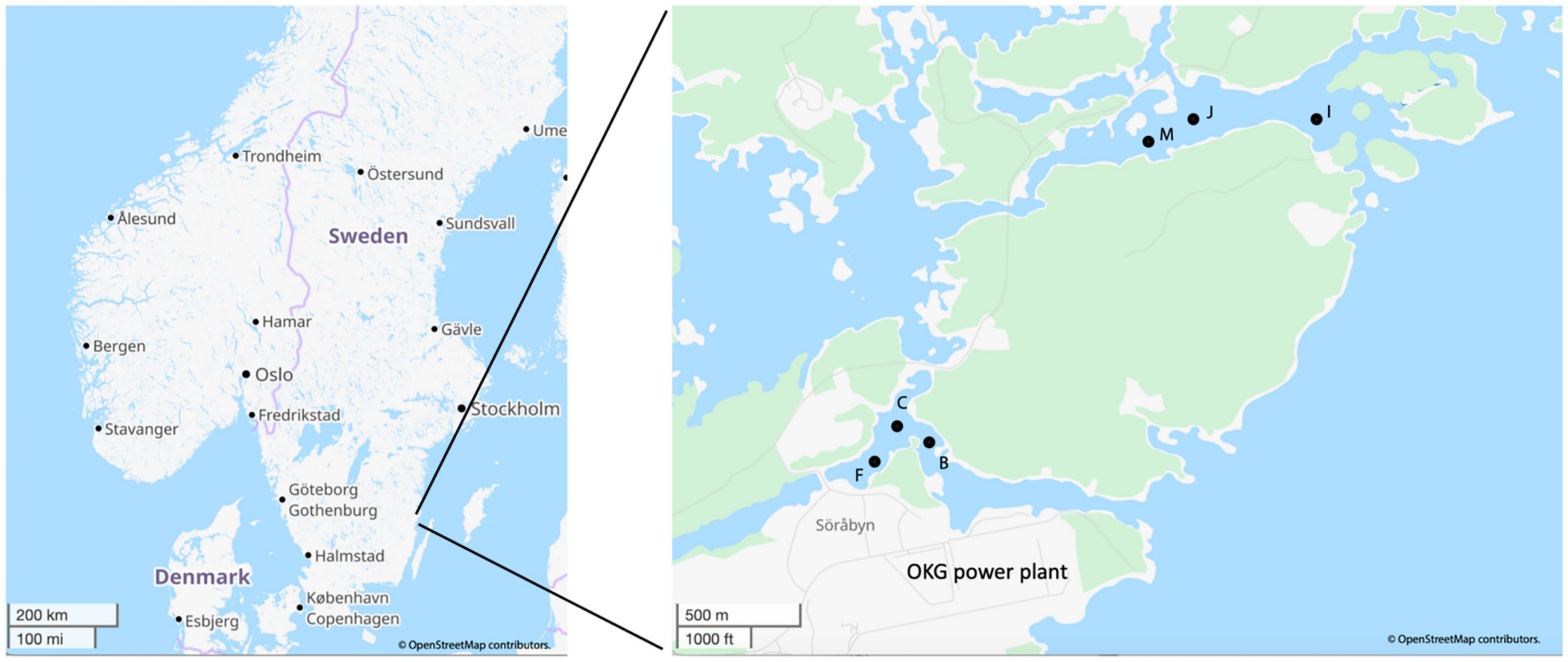
Map of the OKG study area and the locations of the sampling sites within the two bays used in this study. Map created and modified using data from OpenStreetMap, licensed under CC BY-SA 2.0.

The map of the sampling locations is listed in Figure 1 with GPS coordinates, water depths, and geochemical data for all the sampling sites being listed in Supplementary Table S1. Temperature data loggers (HOBOware, Onset Computer Corporation, United States) were installed in both bays 1 meter below the surface to year-round continuously measure water temperature. This long-term temperature monitoring has been previously published (Seidel et al., 2023b) and the data up to and including this study is given in Supplementary Figure S1. While the temperature measurements were made 1 meter below the water surface, due to the shallow depths of the sampling sites, the degree of variation with the bottom water was likely minor and the seasonal trends were consistent. Sediment cores were collected with a HTH gravity corer (Renberg and Hansson, 2008) from both bays in August 2021 as summer samples (*n* = 3 per bay) and in March 2022 as winter samples (*n* = 3 per bay). The sampling details were described in previous studies (Seidel et al., 2022; Seidel et al., 2023b).

All sediment samples were sliced immediately in the field at specific depths for the various purposes (sediment sampling scheme given in Supplementary Figure S2). For gas measurement, sediment cores were sliced at four different depth intervals (2–6 cm, 9–13 cm, 16–20 cm, and 23–27 cm) and 133 mL of sediment was collected at each depth interval, placed into 600 mL gas tight jars (Isojar, www.isotechlabs.com), and 200 mL of distilled water plus 10 drops of benzalkonium chloride were added to retard microbial activity and allow the formation of 200 mL of head space. Porewater was obtained by vacuum filtering 120 cm^3^ of sediment at every 2 cm of depth using Rrhizon filters for summer and every 4 cm for winter. Details of the gas and porewater sampling can be found in published methods (Ketzer et al., 2024). Finally, microbiological nucleic acid samples were collected at five different depths intervals (0–1 cm, 1–2 cm, 8–9 cm, 15–16 cm, and 22–23 cm), transported to the laboratory at 4 °C, and stored in a −80 °C freezer on the same day.

### 2.2 Geochemical analyses and fluxes

Spectrophotometric methods were used to measure sulfate, total iron, and phosphate for sediment pore water based on former research (Broman et al., 2019). Nitrate concentrations were measured by Hach–Lange cuvette test LCK339 as previously described (Seidel et al., 2022). Organic matter content was measured by the loss on ignition (LOI) method by drying the samples at 105 °C for 24 h and heating at 550 °C for 4 h, with the weight loss representing the organic matter content. Methane concentration in the sediment was measured by injecting 0.5 mL of headspace gas from the Isojar samples into a ThermoFisher Trace GC 1310 gas chromatograph equipped with a PoraPLOT-Q 25 m × 0.32 mm capillary column and a flame ionization detector. Operating temperatures were 80 °C (oven), 170 °C (injection), and 250 °C (detector). Helium was used as a carrier gas at a flow rate of 5 mL min^−1^. The concentration of headspace methane was determined by comparing the sample with five calibration standards of differing methane concentrations (10 ppm, 100 ppm, 1,000 ppm, 1, and 10%) diluted in helium. The total CH_4_ concentration (headspace and dissolved in water) was calculated according to the headspace equilibration method for measuring dissolved methane (Magen et al., 2014) and sediment porosity was assumed to be 80% (uncompacted muddy sediment) (Mondol et al., 2007). Sulfate diffusive fluxes were calculated using the Fick’s first law:


J=−D(dC/dX)

where *J* referred to the diffusion flux, D was the diffusion coefficient, d*C* was the difference in methane/sulfate concentration between two depths, and d*X* was the depth difference between the two considered points. The effect on porosity was calculated using a logarithmic equation (Boudreau, 1996). The fluxes were calculated between the seafloor and the depth of no sulfate (Egger et al., 2018) with the latter established considering a linear regression of the sulfate profile. Sulfate profiles in cores F (summer) and I (summer) clearly showed a non-linear profile and were excluded from the calculations.

### 2.3 Nucleic acid extractions, PCR amplification, and sequencing

Procedures for the nucleic acid extractions, polymerase chain reaction (PCR) amplification, and sequencing have been previously described (Seidel et al., 2022; Seidel et al., 2023b). Briefly, sediment DNA extractions were performed using the DNeasy^®^ PowerSoil Extraction Kit (QIAGEN) according to the manufacturer’s instructions. Amplification of 16S rRNA gene fragments were conducted using the PCR primers 341F-805R for bacteria (Herlemann et al., 2011) and 517F-958R for archaea (Dutta et al., 2019). Library preparation was as previously described (Lindh et al., 2015) before sequencing on the Illumina MiSeq platform by Science for Life laboratory (SciLifeLab) in Stockholm, Sweden.

### 2.4 Bioinformatic and statistical analyses

The 16S rRNA gene pair-ends reads (2 × 301 bp) were analyzed using the ampliseq (v. 2.7.0) pipeline (Straub et al., 2020) that included a series of bioinformatic processes (quality control, inference of amplicon sequence variants (ASVs), and taxonomical classification). The Swedish Biodiversity Data Infrastructure (SBDI) Sativa curated 16S Genome Taxonomy Database (GTDB) database (Lundin and Andersson, 2021) was used as reference database for taxonomic assignment. For microbial community dissimilarity, principal coordinate analysis (PCoA) was calculated based on Bray–Curtis dissimilarities by “vegdist” function in “vegan” package (v. 2.6-6.1) (Oksanen, 2010) in R (v. 4.4.1). After that, the permutational analysis of variance (PERMANOVA) was done using the “adonis2” function again from “vegan” package. Relative abundance of each taxonomy level was calculated based on the counts of all related ASVs. Differential abundance analysis was done by “ancombc2” function in “ANCOMBC” package (v. 2.6.0) (Lin and Peddada, 2020) at family level, and *p*-values were adjusted for multiple comparisons using the Holm–Bonferroni method. The canonical correspondence analysis was done by “cca()” function again from “vegan” package. The linear mixed models for geochemical parameters were with the “lmer” function in the “lmerTest” package (v. 4.4.1).

## 3 Results and discussion

The control bay average water temperature during summer (1st June to 22nd September 2021) was 19.9 ± 3.3 °C (range 12.1–27.5 °C) while during winter (21st December – 20th March 2022) it was 2.7 ± 0.9 °C (range 0.01–7.1 °C). The heated bay average water temperature during summer was 23.9 ± 3.4 °C (range 17.3–32.2 °C) compared to 9.5 ± 2.3 °C (range 3.4–14.1 °C) during winter. These temperatures were significantly higher in the heated compared to the control bay (*t*-test, *p* < 0.001; Supplementary Figure S1) and have been consistent between the two bays since the measurements were initiated in December 2017. This also supports the idea that the findings of this study can reflect multi-year patterns, as temperature fluctuations generally follow the same annual trend (Supplementary Figure S1).

Based on the results of the linear mixed-effects model on other geochemical parameters apart from methane and sulfate (including nitrate, phosphate, iron, and organic matter; Supplementary Table S2), the nitrate, phosphate, and organic matter values were significantly correlated with sediment depth (ANOVA, *p* < 0.05). However, no significant differences were observed in those geochemical parameters with respect to the bay factor or the interaction of bay × depth (Supplementary Table S2). This suggested the levels of those geochemical parameters were not statistically different between the bays. Therefore, the major differences were likely associated with methane and sulfate concentrations, which will be discussed in the following sections.

### 3.1 Methane and sulfate concentrations and fluxes in the control bay

**Figure 2:**
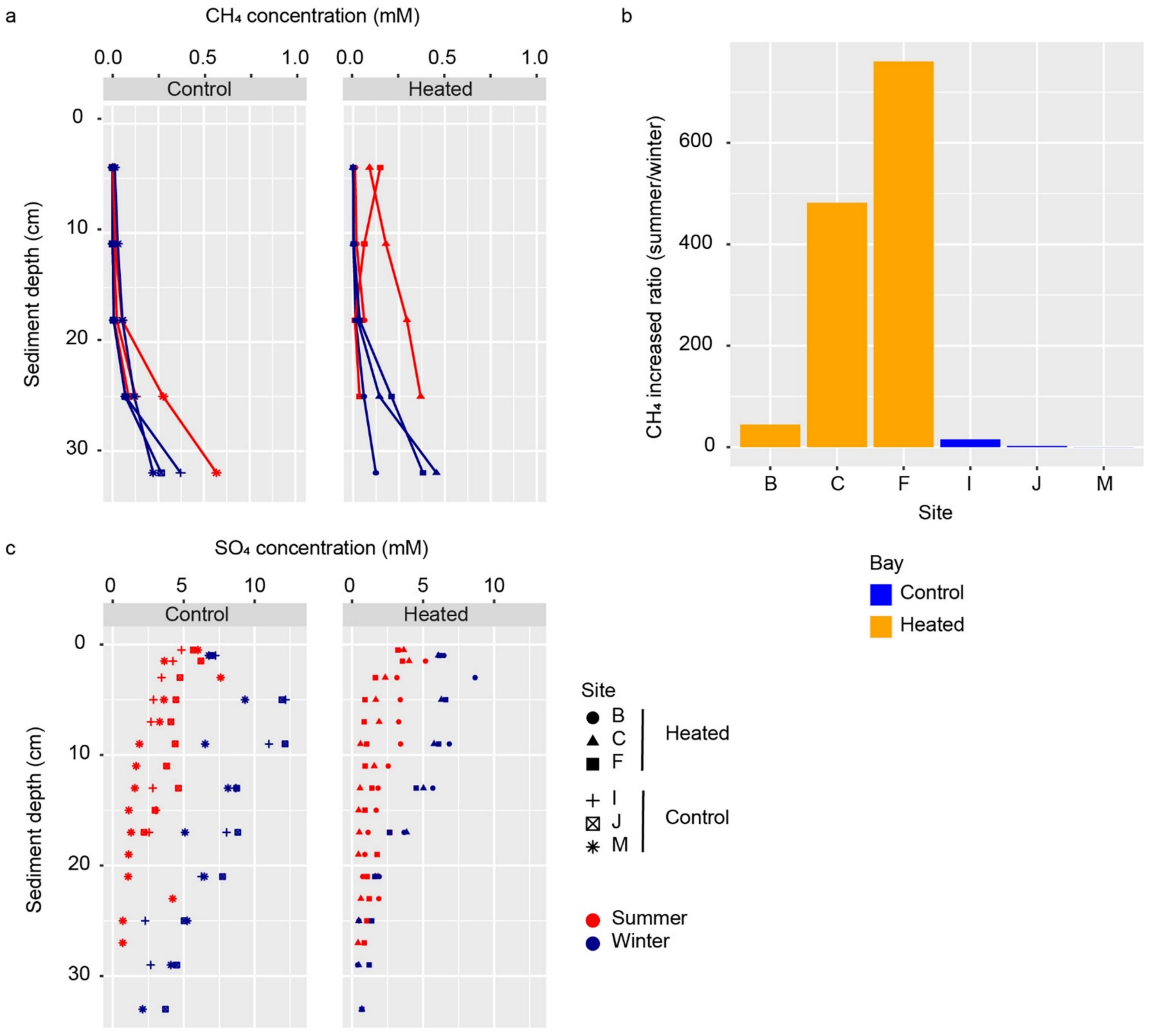
Methane and sulfate concentrations. Methane concentration in the two bays and during the summer and winter sampling occasions **(a)** along with the increased summer methane concentration ratio to the winter value in the two bays at 4 cm sediment depth **(b)**, and sulfate concentration profiles in summer and winter in each bay **(c)**.

Methane concentrations in the control bay sediments increased with depth from 0.001 mM at 4 cm to 0.8 mM at 32 cm below seafloor (Figure 2a). The data also revealed higher methane concentrations in summer compared to winter for all individual sites, notably at depths greater than 20 cm (Figure 2a). The highest increase between winter and summer—approximately 47-fold—was observed at 11 cm below the seafloor at site I. In comparison, near-seafloor methane concentrations (at 4 cm depth) increased by 2-fold and 15-fold at sites J and I, respectively (Figure 2b). The observed variation in the two extreme seasons (winter-summer) in coastal sediment methane concentrations, with higher concentrations in summer and lower concentrations in winter, has been reported in other near-shore regions with different geographical, geological, and climatic contexts (Baltic Sea, Mediterranean Sea, and Wadden Sea) and is attributed to a direct effect of increase temperature on methanogenesis (Sawicka and Brüchert, 2017; de Groot et al., 2023; Flecha et al., 2023). The same temperature increase is believed to have a lesser impact on processes consuming methane (e.g., AOM coupled to sulfate reduction), leading to a methane build up in sediment pores and augmented methane fluxes towards the seafloor (Sawicka and Brüchert, 2017). The seasonal increase in methane concentration in coastal sediments may also lead to an increase in methane emissions to the water column and atmosphere during summer/autumn (Flecha et al., 2023).

The observed methane concentration changes during summer and winter in the control bay were also reflected in the sulfate concentrations as a result of AOM with sulfate (Boetius et al., 2000). Sulfate concentrations decreased from the seafloor down to the depth of no sulfate (DNS); i.e., the depth where all sulfate is consumed in sediments (Borowski et al., 1996) (Figure 2c). The average DNS was shallower in the summer (25 cm) than in the winter (37 cm), indicating a faster sulfate consumption during the warmer season. This observation was also supported by the calculated higher sulfate flux in the summer (average 1.06 mmol cm^−2^ d^−1^) in relation to winter (average 0.68 mmol cm^−2^ d^−1^) in the control bay. The above fluxes and seasonal differences were comparable (though smaller in absolute values) to those calculated for another coastal area of the Baltic Sea of 2.7 and 1.3 mmol cm^−2^ d^−1^ in summer and winter, respectively (Sawicka and Brüchert, 2017). In winter, the sulfate concentration increased from the surface layer to a peak at 5–10 cm depth, likely due to seasonal fluctuations in seawater salinity that lowered the sediment surface sulfate concentrations. However, further investigation is needed to confirm this.

### 3.2 Methane and sulfate concentrations changes in the heated bay

Both summer and winter methane concentrations increased with depth from 0.003 mM at 4 cm to 0.7 mM at 32 cm below seafloor, except for site F in the summer (Figure 2a). The observed pattern of overall increase in methane concentration between summer and winter was also detected in the heated bay, although at a much more pronounced manner. For instance, summer near-seafloor (i.e., 4 cm depth) methane concentrations increased up to 760-fold in relation to winter (compared to a 15-fold increase in the control bay; Figure 2b). Furthermore, with the exception for site F in the heated bay, there were higher methane concentrations in the heated bay in relation to the control bay at all depths and seasons (Figure 2a). The above observations concurred with seasonal changes in temperature having a major impact on the sediment methane concentration (Sawicka and Brüchert, 2017; de Groot et al., 2023; Flecha et al., 2023) and above all, revealed that an average 5 °C increase in the annual average temperature correlated with an increased the near seafloor methane concentrations 50-fold in relation to the control bay. This observation is particularly relevant as climate change may cause an increase in temperature of similar magnitude by the end of this century (Rajendra et al., 2008; Meinshausen et al., 2020). Therefore, it can be anticipated that a 50-fold higher methane concentration than those measured in present-day near seafloor sediments will possibly be encountered in Baltic Sea coastal areas in future climate change scenarios. While there was no direct evidence that warming in the heated bay caused methane release from the seafloor, the increased methane concentration could impair the microbial filter’s ability to remove methane. In addition, other confounding factors such as organic matter input and sediment compaction can also influence the warming effect on methane and sulfate profiles. While the results indicated a correlation between warming and increased methane concentrations, further studies using experimental controls are needed to confirm a direct connection between the two.

Surprisingly, the data revealed little difference between average sulfate fluxes in the heated bay summer (1.01 mmol cm^−2^ d^−1^) plus winter (0.93 mmol cm^−2^ d^−1^) and the control bay summer (1.06 mmol cm^−2^ d^−1^) as compared to 0.68 mmol cm^−2^ d^−1^ for the control bay winter (Supplementary Table S3). A previous study reported higher fluxes in the spring in the heated bay in relation to the control bay (Seidel et al., 2023b) and an average year around higher flux in the heated bay (Seidel et al., 2022). Similar sulfate fluxes during summer but a higher methane concentration in the heated bay may suggest that the AOM cannot consume this excess methane and therefore, it may be at the edge of its capacity to hinder methane accumulation. However, further data such as RNA transcript sequencing would be required to confirm this conclusion. In the ambient control bay conditions, the summer augmented methane production may be compensated or consumed during late autumn and early winter as methane concentrations decrease during late winter (March). However, higher sulfate fluxes occurred even during late winter and probably year-round in the heated bay conditions. Added to the observation that AOM might be at the edge of its capacity, this further implied that the excess methane produced during summer might not be entirely consumed during the colder months, potentially leading to a progressive build up and release of methane from the sea floor. More studies are needed to confirm this hypothesis, but the high methane concentrations found at two sites in the heated bay (C and F; Figure 2a) at 4 cm below the seafloor could indicate an active, albeit small methane diffusive flux from sediments to the water column during summer. It is also important to consider that not only temperature, but an advance in the eutrophication of the Baltic Sea will result in deoxygenation and higher deposition of particulate organic matter and consequently, will potentially increase in the methane generation in sediments (Ketzer et al., 2024).

### 3.3 16S rRNA gene amplicon sequencing

The Illumina 16S rRNA gene amplicon sequencing generated a total of 27,259,341 reads that gave an average of 1,092 amplicon sequence variants (ASVs; min: 13, max: 4,590). Detailed information of sequencing and read numbers are given in Supplementary Table S4 with the rarefaction analyses giving near asymptotic curves suggesting the samples were sequenced to sufficient depth to identify the majority of the microbial community (Supplementary Figure S3).

### 3.4 16S rRNA gene-based community dissimilarity

**Figure 3:**
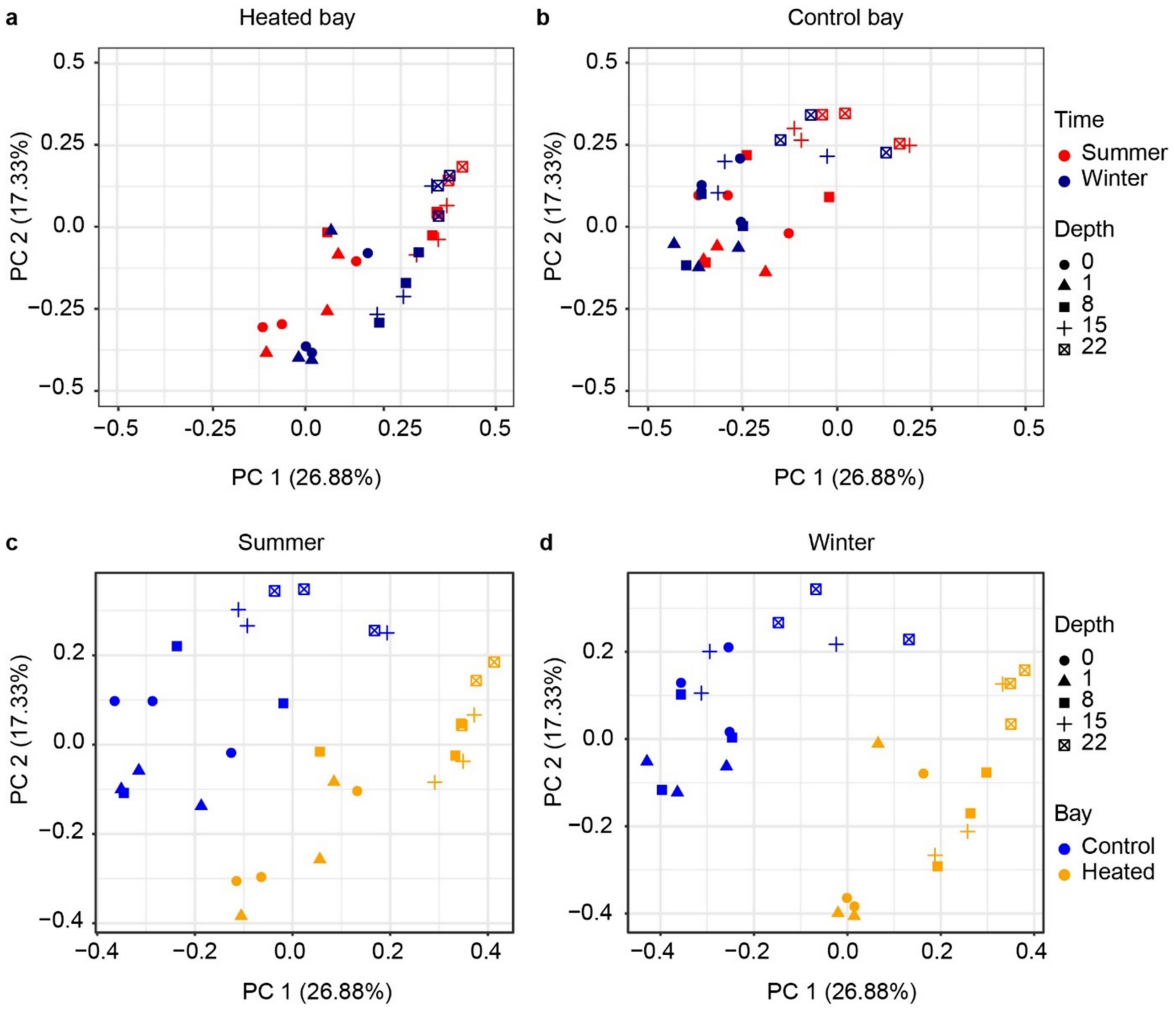
Beta diversity of 16S rRNA gene based archaeal communities. PCoA analysis based on Bray–Curtis dissimilarity of archaea ASVs according to depth between heated **(a)** and control bays **(b)** and in the summer **(c)** and winter **(d)** in each bay.

**Figure 4:**
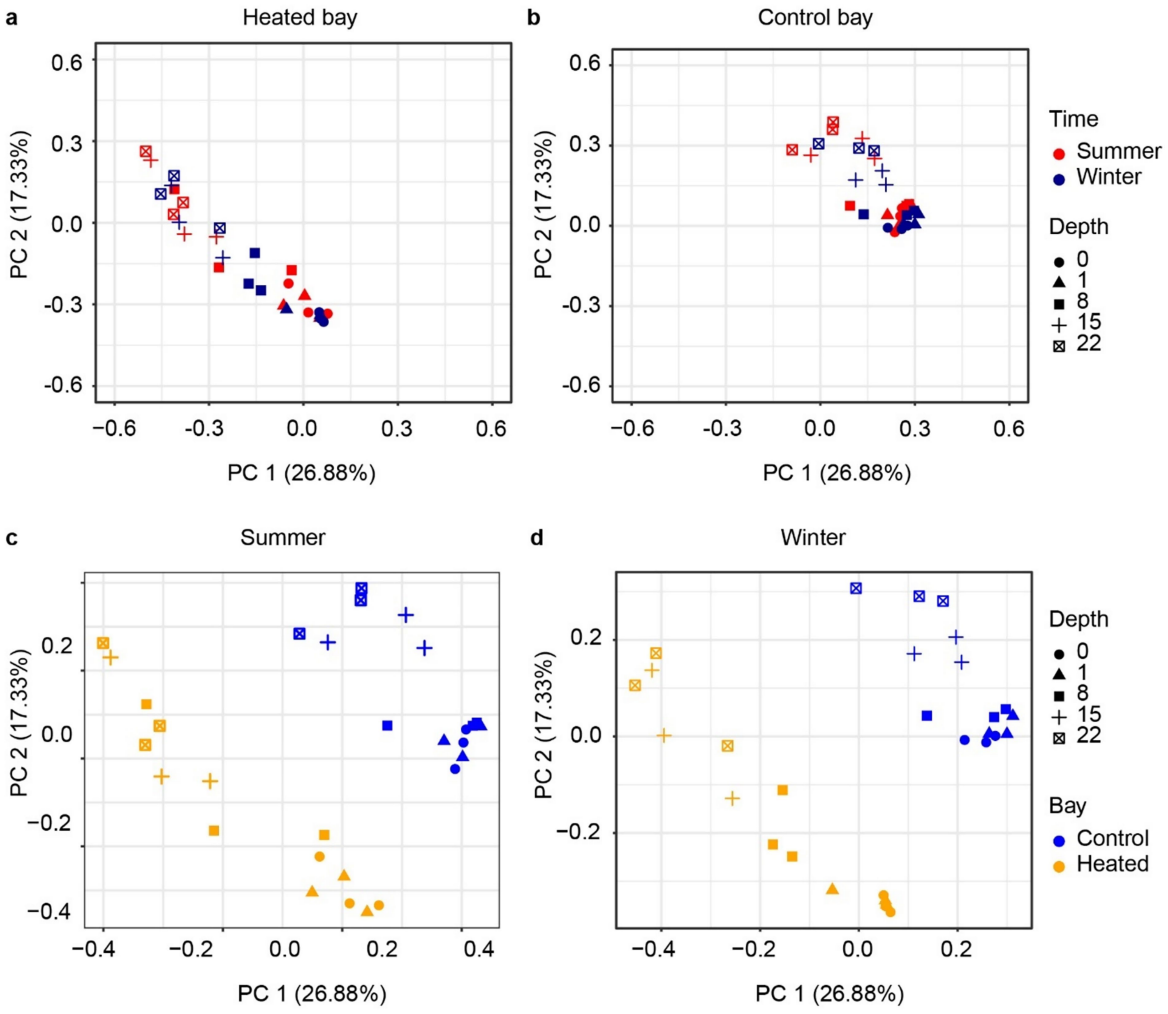
Beta diversity of 16S rRNA gene based bacterial communities. PCoA analysis based on Bray–Curtis dissimilarity of bacteria ASVs according to depth between heated **(a)** and control bays **(b)** and in the summer **(c)** and winter **(d)** in each bay.

The archaeal 16S rRNA gene sequencing ASV dissimilarity showed a clear difference between the heated and control bays in both winter and summer (Figure 3; main effect of bay: *F*_1,48_ = 23.78, *R*^2^ = 0.210, *p* = 0.001), with a longer separation distance in winter (bay × season interaction: *F*_1,48_ = 2.14, *R*^2^ = 0.019, *p* = 0.039). Depth was another important factor separating the archaeal communities and modifying the differentiation between bays (depth: *F*_1,48_ = 15.77, *R*^2^ = 0.139, *p* = 0.001; bay × depth interaction: *F*_1,48_ = 4.54, *R*^2^ = 0.040, *p* = 0.002). The separation distance between the summer and winter was not as clear as the bay-separation in either the heated or control bays, but statistically significant (season: *F*_1,48_ = 2.95, *R*^2^ = 0.026, *p* = 0.006). Similar to the archaeal results, the bacterial community PCoA showed separations among different sediment depths and between the heated and control bays, again with bay modulating the association with sediment depth (Figure 4) (bay: *F*_1,48_ = 25.52, *R*^2^ = 0.206, *p* = 0.001; depth: *F*_1,48_ = 19.34, *R*^2^ = 0.156, *p* = 0.001; bay × depth interaction: *F*_1,48_ = 6.73, *R*^2^ = 0.054, *p* = 0.001). Again, the separation between summer and winter was significant but not as clear as the distance between bays in visualization (season: *F*_1,48_ = 5.86, *R*^2^ = 0.047, *p* = 0.001). In general, bays and depths were two main factors separating the 16S rRNA gene-based microbial communities, which agreed with the previous data (Seidel et al., 2023b).

### 3.5 16S rRNA gene-based winter community

**Figure 5:**
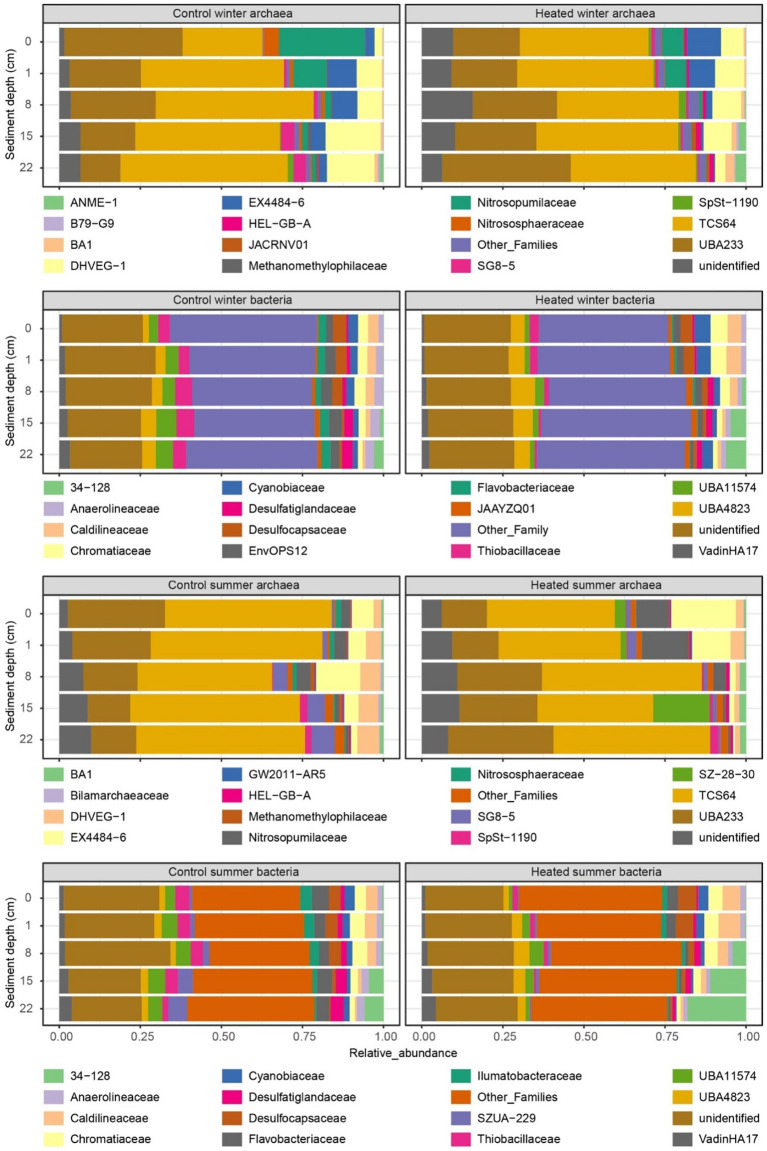
16S rRNA gene-based relative abundances at the level of family. Relative abundance of archaea and bacteria ASVs in each bay and depth during winter and summer. Shown are the families with top 15 highest relative abundance with low relative abundance families listed as “Other_Families”.

**Figure 6:**
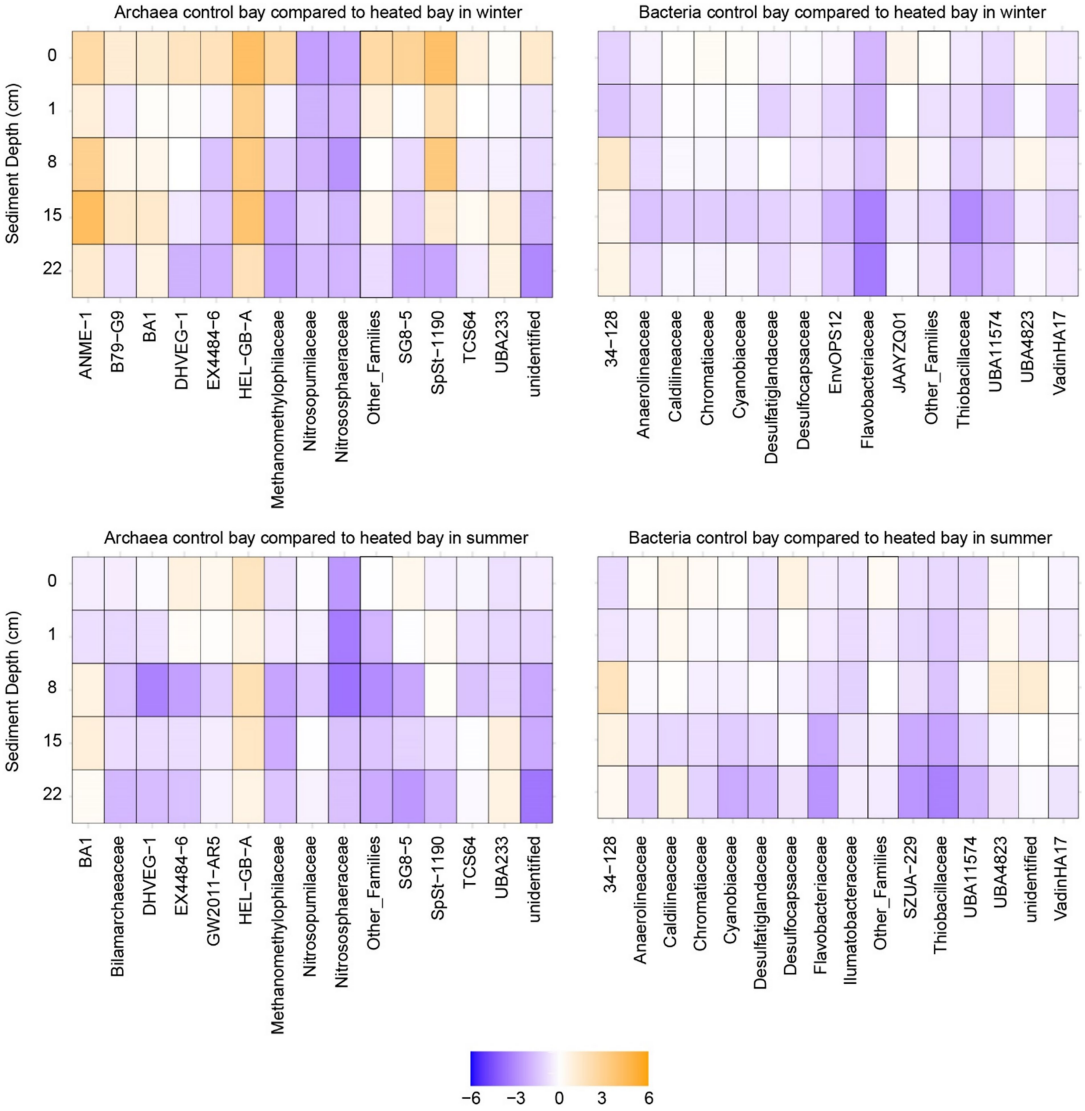
Heatmaps of differential abundance analysis. Differential abundance analysis of bacterial and archaeal populations between the bays plotted against the sediment depth during winter and summer. The analysis was performed by pairwise comparisons between the same depths of the heated and control bays.

The two archaeal families with the highest relative abundance in winter were TCS64 and UBA233 (Figure 5 with further taxonomic levels in Supplementary Figure S4). In the control bay, the UBA233 family had lower mean relative abundance in deep depth sediment (mean ± standard error; 22 cm: 13.0 ± 1.9%) compared to the surface samples (0 cm: 33.8 ± 10.5%), while the mean relative abundance of TCS64 family was increased in deep sediment (22 cm: 51.6 ± 3.7%) compared to the surface (0 cm: 25.7 ± 3.8%). In contrast, the UBA233 family had the opposite condition in the heated bay with increased relative abundance in the deeper sediment (0 cm: 21.6 ± 1.8%; 22 cm: 38.0 ± 6.0%) while family TCS64 maintained a stable mean relative abundance (average around 40%). While these two families lack detailed functional descriptions (Mara et al., 2023; Protasov et al., 2023), they have been previously identified in marine deep sediments and arthropod gut and the Bathyarchaeia class they belong to is widely found in anoxic subsurface environments such as marine and hot-spring sediments (Lloyd et al., 2013; Qi et al., 2021). Bathyarchaeia have diverse capabilities including organic compound metabolism, inorganic carbon assimilation, and anaerobic methane metabolism (Evans et al., 2015; He et al., 2016; Hou et al., 2023). The ANME-1 family had low relative abundance (<1%) in most samples except the deep sediment samples in the heated bay (15 cm: 2.9 ± 1.1%; 22 cm: 3.5 ± 1.6%) with the differential abundance analysis of this family showing significantly increased ASV counts in the heated bay 8 and 15 cm depth sediment samples (*p* < 0.05) (Figure 6 with further statistical results in Supplementary Table S5). ANME-1 is an anaerobic methanotrophic archaea playing an important role in AOM in marine sediments (Laso-Pérez et al., 2023). DHVEG-1 is a further methanotrophic family also found in other sediments (Gründger et al., 2019; Kadnikov et al., 2024) that had low relative abundance at 0 cm (2.9 ± 1.6%) in the control bay, gradually increasing until its peak relative abundance at 22 cm (16.3 ± 1.3%). In contrast, DHVEG-1 had high relative abundance at shallow depths (0 cm: 7.1 ± 1.3%; 1 cm: 10.0 ± 2.8%) then dropped at 22 cm to 3.8 ± 2.3% in the heated bay. BA1 is also a member of the Bathyarchaeia class with genome reconstructions suggesting it contains genes related to methanogenesis (Vanwonterghem et al., 2016). BA1 had low relative abundance (<1%) in the control bay until the deepest depth (22 cm: 1.01 ± 0.02%) compared to the heated bay where its relative abundance increased from 8 cm depth downwards (8 cm: 1.04 ± 0.06%; 15 cm: 1.8 ± 0.4%; 22 cm: 2.9 ± 0.6%). Another methanogenic family, Methanomethylophilaceae (Borrel et al., 2023) was also found in sediment samples, but it had low relative abundance (<1%) at all depths in both bays. In addition, the Nitrosopumilaceae family had a high relative abundance at shallow depths such as the control bay 0 cm sample with 28.1 ± 10.0%. Nitrosopumilaceae are a marine ecosystem associated anaerobic ammonium oxidizer coupled to nitrite reduction (Qin et al., 2017; Rios-Del Toro et al., 2018). The HEL-GB-A family had consistently increased significant ASV counts at all depths except the 22 cm in the heated bay compared to the control bay (0–15 cm: *p* < 0.05). The HEL-GB-A family belongs to taxon Helarchaeales found in other basin sediment samples and has the potential to activate and oxidize hydrothermally generated short-chain hydrocarbons in anaerobic environments (Seitz et al., 2019).

Three sulfate-reducing Desulfobacterota families (Yin et al., 2022) were found in the top 15 relatively abundant bacterial populations. The first was the UBA11574 family with an overall significantly increased abundance in the control bay at 0 cm, 1 cm, and 15 cm depths (*p* < 0.01; Figure 6 with further statistical results in Supplementary Table S5). Its peak relative abundance was also deeper in the control bay (15 cm: 6.0 ± 0.3%) compared to the heated bay (8 cm: 2.8 ± 0.1%). The second Desulfobacterota family was Desulfatiglandaceae with a low relative abundance (<1%) at shallow depths in both bays. However, the peak relative abundance again appeared shallower in the heated bay (at 15 cm: 2.0 ± 0.4%) compared to the control bay (at 22 cm: 3.2 ± 0.2%). Finally, the Desulfocapsaceae had the opposite trend compared to the former two families with the highest relative abundance at 0 cm (control bay: 3.4 ± 0.8%; heated bay: 3.7 ± 0.2%) and lowest at 22 cm (<1% in both bays). The sulfur oxidizing Thibacillaceae family (Watanabe et al., 2019) had deeper relative abundance peak depth in the control bay (lowest at 1 cm: 3.3 ± 0.5%; highest at 15 cm: 5.4 ± 0.5%) compared to the heated bay (highest at 0 cm: 2.8 ± 0.4%), and the differential abundance analysis also showed increased ASV counts at 15 cm and 22 cm depths (*p* < 0.05). Finally, the 34–128 family had a low relative abundance (<1%) at shallow depths in both bays, but with a higher relative abundance in the heated bay in deep depths (15 cm: 4.3 ± 1.4%; 22 cm: 7.1 ± 2.4%) compared to the control bay (15 cm: 1.0 ± 0.1%; 22 cm: 2.8 ± 0.4%). The 34–128 family is a member of the JS1 class in the Atribacteria, which has been found to dominate methanogenic slurry bacterial communities and are likely to be heterotrophic anaerobes predicted to perform primary fermentation of carbohydrates (Nobu et al., 2016; Webster et al., 2023). Unexpectedly, none of the top 15 most abundant families were found to be associated with other electron acceptors (e.g., iron or manganese), likely due to the low abundance of these microbes, which are classified within the “other_families” group.

In summary for winter, there was an increased relative abundance of methanogenic archaea (e.g., BA1) as well as populations suggested to perform AOM (e.g., ANME-1 and DHVEG-1) in shallower depths in the heated compared to the control bay. This supported greater methane concentrations at shallower depths in the heated versus control bay. For the bacterial populations, the sulfate reducing family UBA11574 with increased relative abundance at all depths in the control bay and a deeper peak relative abundance supported the sulfate and methane measurements. This suggested a deeper SMTZ in the control bay that was in concordance with previous data from this study site (Seidel et al., 2023b). Finally, the Atribacteria 34–128 family as a heterotrophic anaerobe increased in relative abundance at a shallower depth in the heated bay, also supporting shallowing of sulfate–methane transition zone in this bay.

### 3.6 16S rRNA gene-based summer community

In summer, the two most dominant archaeal families were also TCS64 and UBA233 from the Bathyarchaeia class, and their relative abundance distributions followed an analogous pattern as for the winter season (Figure 6). Two methanogenic families also had similar trends as for during winter, with the Methanomethylophilaceae family having low relative abundance (<1%) at all depths while the BA1 had increased relative abundances from 8 cm in the heated bay downwards (8 cm: 1.7 ± 0.4%; 15 cm: 1.9 ± 0.7%; 22 cm 1.9 ± 0.8%). However, there was no significant difference in the differential abundance analysis at all depths for BA1 family in summer. Similarly, as for winter, the DHVEG-1 family increased with depth in the control bay (from 0 cm: 2.3 ± 0.1% to 22 cm: 6.7 ± 1.1%). However, its relative abundance decreased at a shallower depth in the heated bay at 8 cm (1.2 ± 0.4%) compared to 22 cm in winter. In contrast to winter, the Nitrosopumilacea family had increased relative abundance at shallow depths in the heated bay that was supported by the differential abundance analysis results (*p* < 0.05). Finally, the HEL-GB-A family had low relative abundance (<1%) at most depths and there was no significant abundance difference between bays in summer.

For sulfate-reducing bacteria from the Desulfobacterota phylum, most populations had similar patterns in summer as for winter. The main difference was for the 34–128 family that had a large increase in relative abundance in the heated bay sediments at 15 cm (16.9 ± 14.4%) and 22 cm (18.1 ± 12.7%) as compared to the shallower depths (e.g., 8 cm: 7.1 ± 5.0%) as well as in the control bay sediment (15 cm: 4.6 ± 1.0%; 22 cm: 4.8 ± 0.9%). The differential abundance analysis results also showed the heated bay had significant increased abundance of 34–128 family ASVs in the heated bay at all depths except 8 cm. As before, the top 15 most abundant families showed no clear connection to taxa known to utilize other electron acceptors.

In general, there were fewer differences in the 16S rRNA gene based microbial communities in summer as compared between the bays in winter. The observed major changes were for the methanotrophic DHVEG-1 family that decreased in relative abundance at a shallower sediment depth in the heated bay while the 34–128 family had increased relative abundances in the heated bay deeper sediment. These findings supported the relative sulfate fluxes and shallowing of the SMTZ in the control bay.

### 3.7 Relationship between microbial community and geochemistry

**Figure 7:**
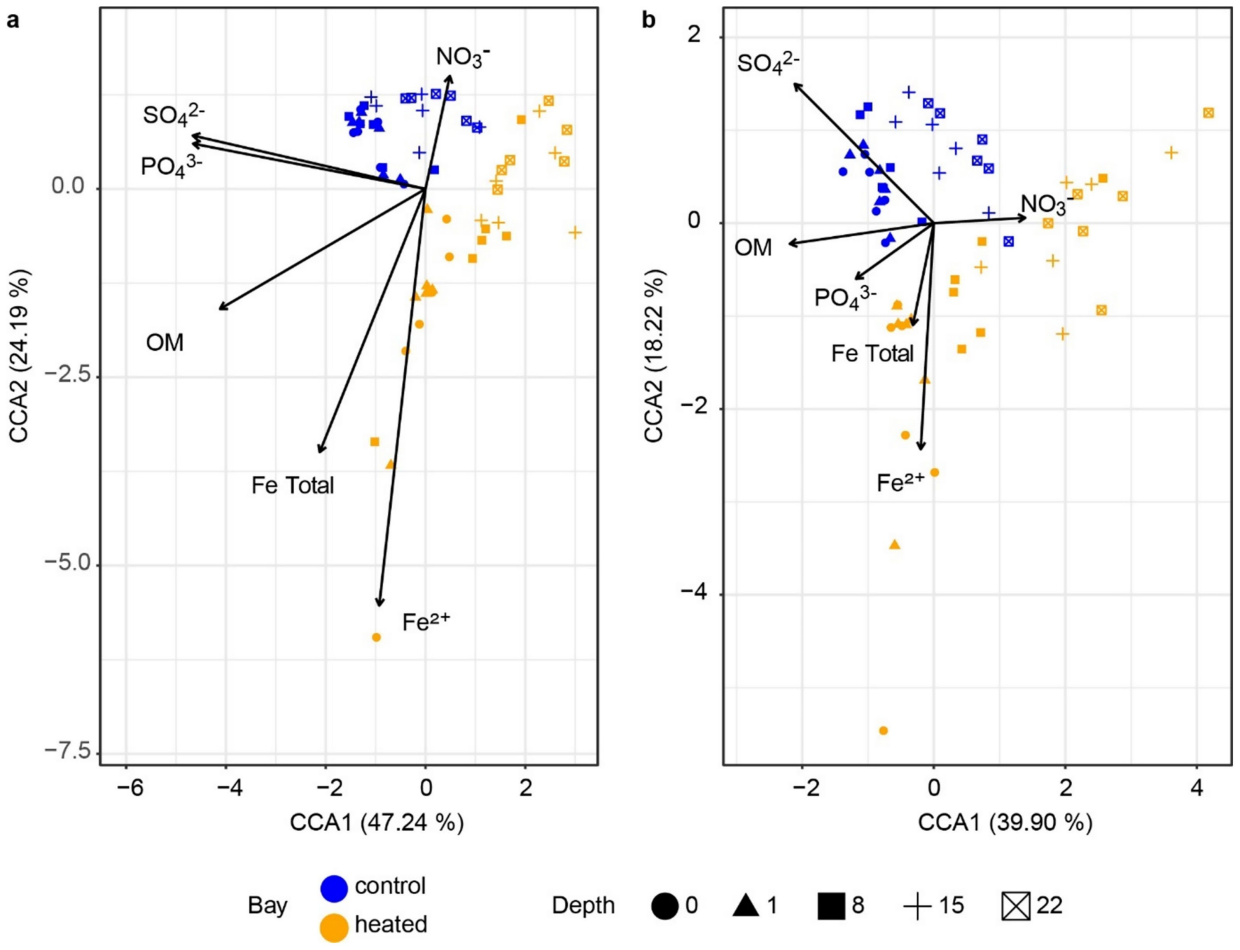
Canonical correspondence analysis plot. Redundancy analysis of the archaea **(a)** and bacteria **(b)** populations with geochemical parameters.

The canonical correspondence analyses of the archaeal and bacterial communities showed that an increased sulfate concentration was associated to the control bay, suggesting lower sulfate fluxes and a deeper sulfate reduction zone in this bay’s sediment (Figure 7). Nitrate, as an important electron acceptor (Kuypers et al., 2018), was driven by both heated and control bay sediment communities and likely reflected that most nitrate had been consumed in the shallow sediments. Organic matter was primarily correlated with microbial communities from shallower depths rather than with bay identity. This pattern was consistent with its significant correlation along the depth gradient but not bay (Supplementary Table S2), suggesting that both bays exhibited similar rates of organic matter degradation. Ferrous iron (Fe^2+^) was mainly associated with shallow heated sediment communities. Since ferrous iron can be treated as an indicator of anaerobic conditions (Sørensen, 1982), it may be a result of SMTZ shallowing in the heated bay. Since methane was sampled at different depths than other parameters, values from the nearest corresponding depths were used for additional canonical correspondence analyses including methane (Supplementary Figure S5). The resulting patterns were similar to other geochemical profiles, with methane oriented toward the deep sediment 16S rRNA gene-based microbial community.

## 4 Conclusion

This study revealed that long-term warming increased the potential for seafloor methane release in Baltic Sea coastal areas and modified the microbial community structure in the sediments. The sediment methane concentration was overall higher in the heated bay in summer. Along with the sulfate flux results, it was shown that long-term warming may accelerate sulfate reduction via anaerobic oxidation of methane, causing shallowing of the SMTZ in the heated bay especially during summer. Correspondingly, the microbial community was also shifted with anaerobic methanotrophic archaea and sulfate reducing bacteria groups peaking at lower sediment depths in the heated bay. Furthermore, both geochemical and microbiology results indicated that future climate change warming could cause a shallowing of the SMTZ in coastal sediment and increase the risks of methane release to the water and atmosphere.

## Data Availability

The 16S rRNA gene amplicon data are available from the European Nucleotide Archive (ENA) (https://www.ebi.ac.uk/ena) under the project accession number PRJEB82349, and sample accession numbers ERS22058692-ERS22058811. The R markdown file used for analysis is available at GitHub: https://github.com/lsjmouse/gas_manu_all_analysis.
