# Supplementary material for: Long-term warming raises risks of seasonal seafloor methane release in the coastal Baltic Sea
Source: Front Microbiol. 2025 Oct 7;16:1636301. doi: 10.3389/fmicb.2025.1636301 (PMC12537722; doi:10.3389/fmicb.2025.1636301)
Supplement: Supplementary file 1 [file Supplementary_file_1.docx]

Supplementary Material

**Long-term warming raises risks of seasonal seafloor**

**methane release in the coastal Baltic Sea**

Songjun Li, Marcelo Ketzer, Cheng Chang, Iryna Rula, Laura Seidel,
Ida Krogsgaard Svendsen, Anders Forsman, Samuel Hylander, & Mark Dopson

**Supplemental Table S1** GPS coordinates, sampling site information, and geochemical data for all the sampling sites.

| **Bay** | **Site** | **GPS coordinates** | **Water depth (m)** | **Distance to shoreline (m)** | **Distance to heating source (m)** |
| --- | --- | --- | --- | --- | --- |
| Heated | B | N 57° 25.268' E 16°40.131' | 3.7 | 40 | 340 |
| Heated | C | N 57° 25.300' E 16°39.996' | 3.2 | 60 | 510 |
| Heated | F | N 57° 25.210' E 16°39.907' | 2.3 | 25 | 450 |
| Control | I | N 57° 25.950' E 16°41.681' | 4.2 | 55 |  |
| Control | J | N 57° 25.974' E 16°41.233' | 6.0 | 80 |  |
| Control | M | N 57° 25.896' E 16°41.029' | 3.3 | 50 |  |

| **Site** | **Season** | **Sediment depth (cm)** | **Methane  conc (mM)** | **Sulfate  conc (mM)** | **Nitrate conc (mg/L)** | **Total Iron (μM)** | **Ferrous Iron (μM)** | **Phosphate (μM)** | **Organic matter (wt %)** |
| --- | --- | --- | --- | --- | --- | --- | --- | --- | --- |
| B | Summer | 0.5 |  | 3.29 | 2.83 | 0.935 | 0.302 | 89.729 | 34.5 |
|  |  | 1.5 |  | 5.17 | 2.35 | 0.260 | 0.049 | 159.168 | 30.6 |
|  |  | 3 |  | 3.16 |  |  |  |  |  |
|  |  | 4 | 0.01 |  |  |  |  |  |  |
|  |  | 5 |  | 3.40 |  |  |  |  |  |
|  |  | 7 |  | 3.29 |  |  |  |  |  |
|  |  | 9 |  | 3.42 | 1.92 | 0.197 | 0.028 | 32.855 |  |
|  |  | 11 | 0.02 | 2.54 |  |  |  |  |  |
|  |  | 13 |  | 1.82 |  |  |  |  |  |
|  |  | 15 |  | 1.70 | 3.36 | 0.226 | 0.113 | 30.144 | 26.6 |
|  |  | 17 |  | 1.13 |  |  |  |  |  |
|  |  | 18 | 0.06 |  |  |  |  |  |  |
|  |  | 19 |  | 0.90 |  |  |  |  |  |
|  |  | 21 |  | 0.75 |  |  |  |  |  |
|  |  | 23 |  | 1.88 | 5.47 | 0.226 | 0.141 | 65.710 | 26.5 |
| C | Summer | 0.5 |  | 3.64 | 3.18 | 3.085 | 2.664 | 227.254 | 36.1 |
|  |  | 1.5 |  | 4.01 | 2.99 | 3.465 | 3.254 | 191.182 | 38.4 |
|  |  | 3 |  | 2.33 |  |  |  |  |  |
|  |  | 4 | 0.09 |  |  |  |  |  |  |
|  |  | 5 |  | 1.67 |  |  |  |  |  |
|  |  | 7 |  | 1.90 |  |  |  |  |  |
|  |  | 9 |  | 0.60 | 3.26 | 1.071 | 0.677 | 120.612 | 29.9 |
|  |  | 11 | 0.18 | 1.56 |  |  |  |  |  |
|  |  | 13 |  | 0.56 |  |  |  |  |  |
|  |  | 15 |  | 0.46 | 3.26 | 0.282 | 0.169 | 57.933 |  |
|  |  | 17 |  | 0.51 |  |  |  |  |  |
|  |  | 18 | 0.29 |  |  |  |  |  |  |
|  |  | 19 |  | 0.45 |  |  |  |  |  |
|  |  | 21 |  | 1.83 |  |  |  |  |  |
|  |  | 23 |  | 0.62 | 4.05 | 0.282 | 0.197 | 54.902 | 26.1 |
|  |  | 25 | 0.37 | 0.46 |  |  |  |  |  |
|  |  | 27 |  | 0.42 |  |  |  |  |  |
| F | Summer | 0.5 |  | 3.22 | 1.96 | 0.555 | 0.513 | 90.631 | 40.9 |
|  |  | 1.5 |  | 3.55 | 2.56 | 0.302 |  | 105.511 | 41.8 |
|  |  | 3 |  | 1.64 |  |  |  |  |  |
|  |  | 4 | 0.15 |  |  |  |  |  |  |
|  |  | 5 |  | 0.90 |  |  |  |  |  |
|  |  | 7 |  | 0.86 |  |  |  |  |  |
|  |  | 9 |  | 1.02 | 2.38 | 0.388 | 0.226 | 76.949 | 31.0 |
|  |  | 11 | 0.06 | 0.92 |  |  |  |  |  |
|  |  | 13 |  | 1.39 |  |  |  |  |  |
|  |  | 15 |  | 0.90 | 2.28 | 0.282 | 0.113 | 28.260 | 30.5 |
|  |  | 17 |  | 2.65 |  |  |  |  |  |
|  |  | 18 | 0.01 |  |  |  |  |  |  |
|  |  | 19 |  | 1.76 |  |  |  |  |  |
|  |  | 21 |  | 1.06 |  |  |  |  |  |
|  |  | 23 |  | 1.21 | 2.28 | 0.226 | 0.113 | 16.860 | 33.1 |
|  |  | 25 | 0.04 | 1.04 |  |  |  |  |  |
|  |  | 27 |  | 0.86 |  |  |  |  |  |
| B | Winter | 1 |  | 6.47 | 1.50 | 0.085 | 0.085 | 13.587 | 39.9 |
|  |  | 2 |  |  | 1.26 | 0.705 | 0.085 | 52.596 | 34.8 |
|  |  | 3 |  | 8.66 |  |  |  |  |  |
|  |  | 4 | 0.00 |  |  |  |  |  |  |
|  |  | 9 |  | 6.84 | 1.24 | 0.648 | 0.197 | 25.421 | 29.5 |
|  |  | 11 | 0.00 |  |  |  |  |  |  |
|  |  | 13 |  | 5.69 |  |  |  |  |  |
|  |  | 15 |  |  | 2.46 | 0.451 | 0.197 | 45.145 | 25.9 |
|  |  | 17 |  | 3.66 |  |  |  |  |  |
|  |  | 18 | 0.01 |  |  |  |  |  |  |
|  |  | 21 |  | 1.91 | 3.11 | 0.620 | 0.282 | 57.417 | 26.9 |
|  |  | 25 | 0.06 | 0.48 |  |  |  |  |  |
|  |  | 29 |  | 0.39 |  |  |  |  |  |
|  |  | 32 | 0.12 |  |  |  |  |  |  |
| C | Winter | 1 |  | 6.07 | 2.90 | 0.226 | 0.056 | 112.703 | 43.4 |
|  |  | 2 |  |  | 2.59 | 0.536 | 0.085 | 125.726 | 40.6 |
|  |  | 4 | 0.00 |  |  |  |  |  |  |
|  |  | 5 |  | 6.29 |  |  |  |  |  |
|  |  | 9 |  | 5.77 | 1.65 | 0.395 | 0.085 | 31.056 | 50.9 |
|  |  | 11 | 0.00 |  |  |  |  |  |  |
|  |  | 13 |  | 5.03 |  |  |  |  |  |
|  |  | 15 |  |  | 1.91 | 0.507 | 0.085 | 34.562 | 29.7 |
|  |  | 17 |  | 3.84 |  |  |  |  |  |
|  |  | 18 | 0.03 |  |  |  |  |  |  |
|  |  | 21 |  | 1.64 | 3.05 | 3.101 | 0.113 | 58.104 | 30.1 |
|  |  | 25 | 0.14 | 0.48 |  |  |  |  |  |
|  |  | 29 |  | 0.47 |  |  |  |  |  |
|  |  | 32 | 0.45 |  |  |  |  |  |  |
|  |  | 33 |  | 0.69 |  |  |  |  |  |
| F | Winter | 1 |  | 6.16 | 3.40 | 0.141 | 0.056 | 24.432 | 46.8 |
|  |  | 2 |  |  | 1.90 | 0.423 | 0.028 | 14.761 | 44.5 |
|  |  | 4 | 0.00 |  |  |  |  |  |  |
|  |  | 5 |  | 6.59 |  |  |  |  |  |
|  |  | 9 |  | 6.10 | 2.23 |  |  | 10.689 | 31.9 |
|  |  | 11 | 0.00 |  |  |  |  |  |  |
|  |  | 13 |  | 4.52 |  |  |  |  |  |
|  |  | 15 |  |  | 2.72 | 0.113 | 0.085 | 24.941 | 30.5 |
|  |  | 17 |  | 2.65 |  |  |  |  |  |
|  |  | 18 | 0.04 |  |  |  |  |  |  |
|  |  | 21 |  | 1.61 | 3.47 | 0.310 | 0.141 | 45.301 | 35.0 |
|  |  | 25 | 0.21 | 1.38 |  |  |  |  |  |
|  |  | 29 |  | 1.21 |  |  |  |  |  |
|  |  | 32 | 0.38 |  |  |  |  |  |  |
|  |  | 33 |  | 0.69 |  |  |  |  |  |
| I | Summer | 0.5 |  | 4.84 | 3.00 | 1.019 | 0.134 | 211.021 | 35.0 |
|  |  | 1.5 |  | 4.24 | 2.91 | 0.387 | 0.091 | 239.428 | 38.0 |
|  |  | 3 |  | 3.44 |  |  |  |  |  |
|  |  | 4 | 0.00 |  |  |  |  |  |  |
|  |  | 5 |  | 2.87 |  |  |  |  |  |
|  |  | 7 |  | 2.70 |  |  |  |  |  |
|  |  | 9 |  |  | 3.15 | 0.423 | 0.254 | 150.008 | 35.9 |
|  |  | 11 | 0.01 |  |  |  |  |  |  |
|  |  | 13 |  | 2.82 |  |  |  |  |  |
|  |  | 15 |  | 3.06 | 3.43 | 0.479 | 0.254 | 29.202 | 30.8 |
|  |  | 17 |  | 2.56 |  |  |  |  |  |
|  |  | 18 | 0.02 |  |  |  |  |  |  |
|  |  | 23 |  |  | 3.72 | 0.254 | 0.169 | 87.757 | 32.7 |
|  |  | 25 | 0.13 |  |  |  |  |  |  |
| J | Summer | 0.5 |  | 5.68 | 1.68 | 0.218 |  | 24.800 | 34.8 |
|  |  | 1.5 |  | 6.21 | 1.66 | 1.061 | 0.049 | 49.599 | 34.2 |
|  |  | 3 |  | 4.74 |  |  |  |  |  |
|  |  | 4 | 0.00 |  |  |  |  |  |  |
|  |  | 5 |  | 4.45 |  |  |  |  |  |
|  |  | 7 |  | 4.10 |  |  |  |  |  |
|  |  | 9 |  | 4.40 | 1.94 | 2.876 | 0.197 | 175.514 | 33.1 |
|  |  | 11 | 0.00 | 3.79 |  |  |  |  |  |
|  |  | 13 |  | 4.63 |  |  |  |  |  |
|  |  | 15 |  | 2.98 | 2.51 | 1.043 | 0.197 | 54.636 | 30.8 |
|  |  | 17 |  | 2.20 |  |  |  |  |  |
|  |  | 18 | 0.01 |  |  |  |  |  |  |
|  |  | 23 |  |  | 2.97 | 0.282 | 0.197 | 47.985 | 31.5 |
|  |  | 25 | 0.09 |  |  |  |  |  |  |
| M | Summer | 0.5 |  | 6.00 | 2.07 | 0.724 | 0.049 | 197.945 | 31.0 |
|  |  | 1.5 |  | 3.64 | 2.65 | 0.597 | 0.218 | 200.651 | 37.7 |
|  |  | 3 |  | 7.60 |  |  |  |  |  |
|  |  | 4 | 0.00 |  |  |  |  |  |  |
|  |  | 5 |  | 3.61 |  |  |  |  |  |
|  |  | 7 |  | 3.31 |  |  |  |  |  |
|  |  | 9 |  | 1.88 | 3.27 | 0.705 | 0.592 | 125.799 | 33.0 |
|  |  | 11 | 0.01 | 1.65 |  |  |  |  |  |
|  |  | 13 |  | 1.56 |  |  |  |  |  |
|  |  | 15 |  | 1.13 | 2.52 | 0.451 | 0.226 | 78.186 | 29.7 |
|  |  | 17 |  | 1.29 |  |  |  |  |  |
|  |  | 18 | 0.05 |  |  |  |  |  |  |
|  |  | 19 |  | 1.12 |  |  |  |  |  |
|  |  | 21 |  | 1.09 |  |  |  |  |  |
|  |  | 23 |  | 4.22 | 2.40 | 0.395 | 0.254 | 89.054 | 29.9 |
|  |  | 25 | 0.28 | 0.72 |  |  |  |  |  |
|  |  | 27 |  | 0.71 |  |  |  |  |  |
|  |  | 32 | 0.57 |  |  |  |  |  |  |
| I | Winter | 1 |  | 7.23 | 1.54 | 0.141 | 0.056 | 19.389 | 45.9 |
|  |  | 2 |  |  | 2.29 | 0.282 | 0.056 | 64.930 | 49.3 |
|  |  | 4 | 0.00 |  |  |  |  |  |  |
|  |  | 5 |  | 12.17 |  |  |  |  |  |
|  |  | 9 |  | 11.01 | 2.29 | 1.241 | 0.141 | 138.426 | 38.2 |
|  |  | 11 | 0.00 |  |  |  |  |  |  |
|  |  | 13 |  | 8.67 |  |  |  |  |  |
|  |  | 15 |  |  | 2.30 | 0.930 | 0.226 | 35.621 | 32.7 |
|  |  | 17 |  | 8.02 |  |  |  |  |  |
|  |  | 18 | 0.01 |  |  |  |  |  |  |
|  |  | 21 |  | 6.26 | 3.09 | 0.874 | 0.226 | 81.613 | 33.2 |
|  |  | 25 | 0.07 | 2.29 |  |  |  |  |  |
|  |  | 29 |  | 2.67 |  |  |  |  |  |
|  |  | 32 | 0.37 |  |  |  |  |  |  |
| J | Winter | 1 |  | 6.93 | 1.96 | 0.169 | 0.056 | 38.327 | 32.7 |
|  |  | 2 |  |  | 2.10 | 0.254 | 0.056 | 190.731 | 41.9 |
|  |  | 4 | 0.00 |  |  |  |  |  |  |
|  |  | 5 |  | 11.92 |  |  |  |  |  |
|  |  | 9 |  | 12.14 | 3.59 | 2.115 | 0.141 | 250.700 | 34.6 |
|  |  | 11 | 0.00 |  |  |  |  |  |  |
|  |  | 13 |  | 8.73 |  |  |  |  |  |
|  |  | 15 |  |  | 2.10 | 1.297 | 0.169 | 167.735 | 32.4 |
|  |  | 17 |  | 8.80 |  |  |  |  |  |
|  |  | 18 | 0.01 |  |  |  |  |  |  |
|  |  | 21 |  | 7.74 | 3.27 | 0.620 | 0.141 | 103.707 | 31.4 |
|  |  | 25 | 0.07 | 5.03 |  |  |  |  |  |
|  |  | 29 |  | 4.50 |  |  |  |  |  |
|  |  | 32 | 0.27 |  |  |  |  |  |  |
|  |  | 33 |  | 3.72 |  |  |  |  |  |
| M | Winter | 1 |  | 6.77 | 2.07 | 0.169 | 0.028 | 17.585 | 46.2 |
|  |  | 2 |  |  | 2.93 | 1.015 | 0.141 | 79.809 | 37.8 |
|  |  | 4 | 0.01 |  |  |  |  |  |  |
|  |  | 5 |  | 9.32 |  |  |  |  |  |
|  |  | 9 |  | 6.51 | 2.66 | 0.620 | 0.310 | 137.074 | 33.4 |
|  |  | 11 | 0.03 |  |  |  |  |  |  |
|  |  | 13 |  | 8.12 |  |  |  |  |  |
|  |  | 15 |  |  | 2.90 | 0.423 | 0.282 | 74.399 | 33.2 |
|  |  | 17 |  | 5.09 |  |  |  |  |  |
|  |  | 18 | 0.05 |  |  |  |  |  |  |
|  |  | 21 |  | 6.46 | 3.20 | 0.451 | 0.169 | 114.078 | 28.9 |
|  |  | 25 | 0.12 | 5.25 |  |  |  |  |  |
|  |  | 29 |  | 4.10 |  |  |  |  |  |
|  |  | 32 | 0.22 |  |  |  |  |  |  |
|  |  | 33 |  | 2.11 |  |  |  |  |  |

**Supplemental Table S2** Statistical analysis (mixed model ANOVA, with site settled as random effect) at geochemical data between bays in both summer and winter.

| Nitrate | *DFnum, DFden* | *F* | *P* |
| --- | --- | --- | --- |
| Bay | 1, 56 | 0.015 | 0.903 |
| Depth | 1, 56 | 15.392 | 0.0002 |
| Bay*Depth | 1, 56 | 0.160 | 0.691 |
| Total Iron | *DFnum, DFden* | *F* | *P* |
| Bay | 1, 7.28 | 0.042 | 0.843 |
| Depth | 1,50.99 | 0.239 | 0.627 |
| Bay*Depth | 1,50.99 | 0.336 | 0.565 |
| Ferrous Iron | *DFnum, DFden* | *F* | *P* |
| Bay | 1, 8.68 | 3.249 | 0.106 |
| Depth | 1, 51 | 1.180 | 0.282 |
| Bay*Depth | 1, 51 | 3.547 | 0.065 |
| Phosphate | *DFnum, DFden* | *F* | *P* |
| Bay | 1, 14.04 | 1.670 | 0.217 |
| Depth | 1, 52 | 5.504 | 0.023 |
| Bay*Depth | 1, 52 | 0.370 | 0.546 |
| Organic matter | *DFnum, DFden* | *F* | *P* |
| Bay | 1, 9.12 | 0.062 | 0.809 |
| Depth | 1, 52 | 17.972 | <0.0001 |
| Bay*Depth | 1, 52 | 0.847 | 0.362 |

**Supplemental Table S3** Diffusive sulfate fluxes values and related information for calculation. Flux unit (mmol m⁻² d⁻¹) is the amount of a substance, expressed in millimoles, that passes through one square meter of surface area per day.

| Bay | Season | Site | Temperature (°C) | Salinity (PSU) | Estimated DNS (cm) | Flux (mmol.m^-2^.d^-1^) |
| --- | --- | --- | --- | --- | --- | --- |
| Heated | Summer | B | 18.2 | 6.8 | 23.959 | 0.99 |
|  |  | C | 18.2 | 6.7 | 21.003 | 1.03 |
|  |  | F | 19.6 | 6.6 |  |  |
| Average flux | | | | | | 1.01 |
|  | Winter | B | 10.3 | 7.2 | 27.558 | 1.05 |
|  |  | C | 10.3 | 7.2 | 30.828 | 0.88 |
|  |  | F | 10.3 | 7.2 | 31.93 | 0.86 |
| Average flux | | | | | | 0.93 |
| Control | Summer | I | 10.8 | 6.6 |  |  |
|  |  | J | 10.5 | 5.8 | 26.839 | 1.01 |
|  |  | M | 11.8 | 5.7 | 23.302 | 1.11 |
| Average flux | | | | | | 1.06 |
|  | Winter | I | 3.7 | 7.2 | 31.039 | 0.82 |
|  |  | J | 3.7 | 7.2 | 38.111 | 0.64 |
|  |  | M | 4.4 | 7.2 | 42.023 | 0.58 |
| Average flux | | | | | | 0.68 |

**Supplemental Table S4** Information of 16S rRNA gene amplicon sequencing.

| **Site** | **Depth** | **Primers/target** | **Kreads** | **>=Q30** | **Input sequences** | **Filtered sequences** | **Unique ASVs** |
| --- | --- | --- | --- | --- | --- | --- | --- |
| Winter bacteria sequencing | | | | | | | |
| M | 0 | 341F-805R/bacteria | 160 | 81.92 | 153863 | 125197 | 1784 |
| J | 0 |  | 160 | 81.87 | 158275 | 128815 | 1934 |
| I | 0 |  | 520 | 81.39 | 506496 | 407553 | 4590 |
| B | 0 |  | 180 | 81.59 | 171838 | 138411 | 2053 |
| C | 0 |  | 200 | 81.31 | 191138 | 153429 | 1848 |
| F | 0 |  | 210 | 81.33 | 209547 | 168475 | 2252 |
| M | 1 |  | 230 | 81.86 | 220508 | 179207 | 2540 |
| J | 1 |  | 270 | 81.72 | 258187 | 209372 | 2557 |
| I | 1 |  | 420 | 81.25 | 406069 | 324850 | 3916 |
| B | 1 |  | 210 | 81.01 | 209670 | 166294 | 2073 |
| C | 1 |  | 220 | 81.02 | 211332 | 168725 | 2006 |
| F | 1 |  | 110 | 80.96 | 107089 | 85360 | 1375 |
| M | 8 |  | 90 | 82.07 | 88495 | 72148 | 1267 |
| J | 8 |  | 360 | 82.13 | 346773 | 282962 | 2954 |
| I | 8 |  | 520 | 81.67 | 497157 | 401454 | 3752 |
| B | 8 |  | 410 | 81.39 | 397351 | 314508 | 4416 |
| C | 8 |  | 110 | 81.21 | 104916 | 83416 | 1460 |
| F | 8 |  | 150 | 81.28 | 146268 | 116802 | 2034 |
| M | 15 |  | 470 | 81.13 | 449735 | 359979 | 3916 |
| J | 15 |  | 140 | 81.38 | 132342 | 106290 | 1511 |
| I | 15 |  | 340 | 80.75 | 320819 | 253990 | 2940 |
| B | 15 |  | 130 | 80.21 | 127452 | 98696 | 1814 |
| C | 15 |  | 100 | 80.29 | 96788 | 75187 | 1243 |
| F | 15 |  | 190 | 80.06 | 183177 | 141149 | 2221 |
| M | 22 |  | 240 | 81.73 | 230697 | 186718 | 2704 |
| J | 22 |  | 200 | 82.10 | 186335 | 152062 | 1991 |
| I | 22 |  | 170 | 81.35 | 156728 | 125621 | 1868 |
| B | 22 |  | 130 | 80.74 | 128763 | 100291 | 1639 |
| C | 22 |  | 350 | 80.88 | 337891 | 262957 | 3021 |
| F | 22 |  | 90 | 80.78 | 89027 | 69559 | 1119 |
| Winter archaea sequencing | | | | | | | |
| J | 0 | 517F-958R/archaea | 93 | 90.24 | 88674 | 82790 | 224 |
| J | 1 |  | 586 | 90.76 | 569268 | 531296 | 521 |
| J | 8 |  | 292 | 81.34 | 143776 | 133827 | 315 |
| J | 15 |  | 275 | 84.09 | 230226 | 214242 | 518 |
| J | 22 |  | 172 | 91.14 | 167727 | 157058 | 432 |
| M | 0 |  | 150 | 92.15 | 148493 | 139603 | 294 |
| M | 1 |  | 355 | 70.81 | 208535 | 193440 | 405 |
| M | 8 |  | 194 | 91.34 | 191976 | 179678 | 398 |
| M | 15 |  | 101 | 90.09 | 95913 | 89850 | 373 |
| M | 22 |  | 162 | 88.19 | 150196 | 140179 | 464 |
| I | 0 |  | 172 | 91.66 | 170354 | 159459 | 220 |
| I | 1 |  | 383 | 91.26 | 374198 | 349224 | 483 |
| I | 8 |  | 571 | 90.45 | 553446 | 514243 | 503 |
| I | 15 |  | 168 | 89.88 | 159790 | 149391 | 374 |
| I | 22 |  | 87 | 87.92 | 79784 | 74544 | 293 |
| B | 0 |  | 511 | 86.19 | 456702 | 425173 | 824 |
| B | 1 |  | 630 | 89.32 | 595922 | 556259 | 814 |
| B | 8 |  | 303 | 90.11 | 294417 | 274026 | 771 |
| B | 15 |  | 541 | 86.61 | 487100 | 453108 | 742 |
| B | 22 |  | 147 | 91.17 | 144676 | 135010 | 392 |
| C | 0 |  | 200 | 83.85 | 167645 | 156042 | 516 |
| C | 1 |  | 85 | 72.73 | 52703 | 49065 | 247 |
| C | 8 |  | 395 | 89.30 | 377153 | 351371 | 684 |
| C | 15 |  | 584 | 87.50 | 536583 | 498920 | 827 |
| C | 22 |  | 190 | 59.35 | 70331 | 64090 | 356 |
| F | 0 |  | 352 | 79.55 | 267542 | 248112 | 583 |
| F | 1 |  | 568 | 77.46 | 406435 | 375426 | 750 |
| F | 8 |  | 181 | 78.09 | 137722 | 123096 | 527 |
| F | 15 |  | 67 | 78.17 | 50095 | 46373 | 279 |
| F | 22 |  | 181 | 76.44 | 127540 | 118564 | 345 |
| Summer bacteria sequencing | | | | | | | |
| F | 0 | 341F-805R/bacteria | 265 | 71.35 | 257448 | 140271 | 1331 |
| B | 0 |  | 167 | 71.55 | 163571 | 92022 | 1018 |
| C | 0 |  | 254 | 71.59 | 249007 | 140984 | 1429 |
| I | 0 |  | 167 | 71.45 | 160790 | 90552 | 919 |
| J | 0 |  | 196 | 71.36 | 187336 | 104102 | 951 |
| M | 0 |  | 231 | 71.14 | 221743 | 123140 | 1178 |
| F | 1 |  | 181 | 71.98 | 176649 | 102846 | 1076 |
| B | 1 |  | 227 | 72.09 | 221793 | 128351 | 1504 |
| C | 1 |  | 192 | 72.31 | 188791 | 112423 | 1123 |
| I | 1 |  | 154 | 70.37 | 147796 | 75348 | 656 |
| J | 1 |  | 284 | 71.02 | 273176 | 145142 | 1200 |
| M | 1 |  | 461 | 70.98 | 444438 | 235366 | 1829 |
| F | 8 |  | 116 | 71.60 | 114167 | 64834 | 737 |
| B | 8 |  | 144 | 71.12 | 141000 | 76102 | 887 |
| C | 8 |  | 398 | 71.96 | 389475 | 224903 | 2243 |
| I | 8 |  | 195 | 71.97 | 189003 | 110510 | 1070 |
| J | 8 |  | 228 | 72.13 | 221876 | 130276 | 1266 |
| M | 8 |  | 153 | 71.02 | 147117 | 81201 | 811 |
| F | 15 |  | 142 | 71.63 | 138905 | 79614 | 958 |
| B | 15 |  | 113 | 70.79 | 110542 | 59110 | 562 |
| C | 15 |  | 347 | 70.35 | 336554 | 171099 | 1630 |
| I | 15 |  | 215 | 71.74 | 205918 | 119726 | 1120 |
| J | 15 |  | 245 | 71.73 | 234586 | 136045 | 1205 |
| M | 15 |  | 280 | 70.92 | 267723 | 147262 | 1240 |
| F | 22 |  | 111 | 70.56 | 108241 | 57043 | 639 |
| B | 22 |  | 192 | 71.20 | 188592 | 105668 | 938 |
| C | 22 |  | 232 | 69.88 | 223350 | 113802 | 964 |
| I | 22 |  | 177 | 71.46 | 171314 | 97536 | 879 |
| J | 22 |  | 293 | 71.87 | 283040 | 165183 | 1293 |
| M | 22 |  | 345 | 70.76 | 332219 | 178837 | 1684 |
| Summer archaea sequencing | | | | | | | |
| C | 0 | 517F-958R/archaea | 175 | 81.09 | 173337 | 145949 | 274 |
| B | 0 |  | 161 | 82.07 | 159920 | 136937 | 424 |
| F | 0 |  | 175 | 81.21 | 173980 | 146404 | 407 |
| M | 0 |  | 160 | 81.70 | 158870 | 135392 | 310 |
| J | 0 |  | 145 | 81.70 | 143644 | 122209 | 268 |
| I | 0 |  | 151 | 81.91 | 149691 | 128245 | 238 |
| C | 1 |  | 196 | 81.79 | 193832 | 165762 | 352 |
| B | 1 |  | 172 | 81.78 | 170190 | 144336 | 448 |
| F | 1 |  | 194 | 80.89 | 192925 | 161049 | 468 |
| M | 1 |  | 294 | 81.13 | 290566 | 243737 | 454 |
| J | 1 |  | 345 | 81.47 | 341415 | 289018 | 379 |
| I | 1 |  | 413 | 81.51 | 408443 | 346794 | 362 |
| C | 8 |  | <1 | 72.91 | 601 | 369 | 13 |
| B | 8 |  | 330 | 81.93 | 327628 | 280179 | 524 |
| F | 8 |  | 171 | 79.36 | 169945 | 134530 | 476 |
| M | 8 |  | 193 | 81.25 | 190635 | 160211 | 471 |
| J | 8 |  | 95 | 81.5 | 93782 | 79353 | 283 |
| I | 8 |  | 540 | 81.34 | 532464 | 450271 | 531 |
| C | 15 |  | 164 | 80.79 | 162373 | 134892 | 445 |
| B | 15 |  | 322 | 81.00 | 320275 | 268089 | 520 |
| F | 15 |  | 598 | 79.94 | 594425 | 481044 | 749 |
| M | 15 |  | 162 | 80.04 | 160593 | 129492 | 321 |
| J | 15 |  | 171 | 81.28 | 169401 | 142726 | 386 |
| I | 15 |  | 191 | 81.53 | 190052 | 160522 | 365 |
| C | 22 |  | 158 | 79.02 | 156991 | 123009 | 322 |
| B | 22 |  | 162 | 80.95 | 160501 | 134265 | 338 |
| F | 22 |  | 174 | 80.54 | 172381 | 142795 | 331 |
| M | 22 |  | 154 | 80.73 | 152768 | 126047 | 326 |
| J | 22 |  | 154 | 81.56 | 153279 | 129317 | 334 |
| I | 22 |  | 170 | 82.05 | 168626 | 144419 | 337 |

**Supplemental Table S5** Information of pair-wise comparison output from the differential abundance analysis based on 16S rRNA ASVs results.

| Family | lfc_0cm | raw_p_value_0cm | adjusted_p_value_0cm | lfc_1cm | raw_p_value_1cm | adjusted_p_value_1cm | lfc_8cm | raw_p_value_8cm | adjusted_p_value_8cm | lfc_15cm | raw_p_value_15cm | adjusted_p_value_15cm | lfc_22cm | raw_p_value_22cm | adjusted_p_value_22cm |
| --- | --- | --- | --- | --- | --- | --- | --- | --- | --- | --- | --- | --- | --- | --- | --- |
| **Summer archaea** | | | | | | | | | | | | | | | |
| EX4484-6 | 0.824 | 0.287 | 1 | 0.192 | 0.805 | 1 | -2.465 | 0.016 | 0.558 | -0.826 | 0.253 | 1 | -1.616 | 0.074 | 1 |
| UBA233 | -0.795 | 0.069 | 1 | -1.01 | 0.1 | 1 | -1.102 | 0.432 | 1 | 0.909 | 0.062 | 1 | 0.72 | 0.23 | 1 |
| unidentified | -0.47 | 0.7 | 1 | -1.036 | 0.241 | 1 | -2.214 | 0.045 | 1 | -2.17 | 0.013 | 0.502 | -3.557 | 0.007 | 0.282 |
| Nitrososphaeraceae | -2.669 | 0.036 | 1 | -3.394 | 0.005 | 0.198 | -3.64 | 0.006 | 0.258 | -1.576 | 0.12 | 1 | -1.551 | 0.111 | 1 |
| DHVEG-1 | -0.128 | 0.832 | 1 | -0.824 | 0.321 | 1 | -3.198 | 0.031 | 1 | -0.898 | 0.102 | 1 | -1.688 | 0.009 | 0.395 |
| TCS64 | -0.256 | 0.558 | 1 | -0.843 | 0.202 | 1 | -1.574 | 0.27 | 1 | -0.043 | 0.929 | 1 | -0.17 | 0.736 | 1 |
| SG8-5 | 0.418 | 0.508 | 1 | -0.058 | 0.945 | 1 | -2.271 | 0.053 | 1 | -1.108 | 0.066 | 1 | -2.667 | 0 | 0.012 |
| Nitrosopumilaceae | -0.065 | 0.968 | 1 | -0.386 | 0.81 | 1 | -1.403 | 0.409 | 1 | -0.037 | 0.972 | 1 | -0.321 | 0.707 | 1 |
| Bilamarchaeaceae | -0.42 | 0.761 | 1 | -0.985 | 0.307 | 1 | -1.626 | 0.166 | 1 | -0.861 | 0.486 | 1 | -1.814 | 0.058 | 1 |
| GW2011-AR5 | 0.437 | 0.712 | 1 | 0.102 | 0.899 | 1 | -1.206 | 0.276 | 1 | -0.437 | 0.592 | 1 | -0.38 | 0.658 | 1 |
| SpSt-1190 | -0.401 | 0.781 | 1 | 0.332 | 0.765 | 1 | 0.141 | 0.902 | 1 | -0.808 | 0.457 | 1 | -1.793 | 0.237 | 1 |
| BA1 | -0.431 | 0.731 | 1 | -0.807 | 0.371 | 1 | 0.766 | 0.467 | 1 | 0.966 | 0.245 | 1 | 0.262 | 0.763 | 1 |
| HEL-GB-A | 1.643 | 0.294 | 1 | 0.776 | 0.47 | 1 | 1.973 | 0.177 | 1 | 1.597 | 0.091 | 1 | 0.692 | 0.502 | 1 |
| Methanomethylophilaceae | -0.745 | 0.639 | 1 | -0.594 | 0.577 | 1 | -2.331 | 0.047 | 1 | -2.121 | 0.046 | 1 | -1.465 | 0.146 | 1 |
| **winter archaea** | | | | | | | | | | | | | | | |
| EX4484-6 | 1.821 | 0 | 0.01 | -0.314 | 0.624 | 1 | -1.54 | 0.021 | 0.42 | -1.48 | 0.029 | 0.551 | -2.005 | 0.002 | 0.052 |
| ANME-1 | 2.347 | 0.079 | 1 | 0.963 | 0.344 | 1 | 3.002 | 0.001 | 0.05 | 4.312 | 0 | 0.005 | 1.258 | 0.353 | 1 |
| unidentified | 1.382 | 0.032 | 0.886 | -0.698 | 0.324 | 1 | -0.954 | 0.169 | 1 | -1.987 | 0.018 | 0.625 | -3.053 | 0 | 0.015 |
| Nitrososphaeraceae | -2.282 | 0.009 | 0.315 | -1.868 | 0.034 | 0.977 | -2.78 | 0.002 | 0.075 | -1.84 | 0.05 | 1 | -1.905 | 0.024 | 0.773 |
| UBA233 | 0.14 | 0.754 | 1 | -0.117 | 0.842 | 1 | -0.39 | 0.512 | 1 | 0.948 | 0.104 | 1 | 0.802 | 0.214 | 1 |
| SG8-5 | 2.809 | 0 | 0 | -0.085 | 0.904 | 1 | -0.921 | 0.246 | 1 | -1.357 | 0.035 | 1 | -2.353 | 0.001 | 0.039 |
| TCS64 | 0.979 | 0.001 | 0.055 | 0.026 | 0.959 | 1 | -0.459 | 0.475 | 1 | 0.347 | 0.569 | 1 | -0.542 | 0.307 | 1 |
| Nitrosopumilaceae | -2.46 | 0.048 | 1 | -1.99 | 0.192 | 1 | -1.975 | 0.077 | 1 | -1.251 | 0.224 | 1 | -1.716 | 0.151 | 1 |
| DHVEG-1 | 1.675 | 0.006 | 0.248 | 0.12 | 0.851 | 1 | 0.03 | 0.967 | 1 | -0.527 | 0.486 | 1 | -1.987 | 0.01 | 0.372 |
| SpSt-1190 | 4.064 | 0 | 0.001 | 1.96 | 0.032 | 0.882 | 3.538 | 0 | 0.008 | 1.11 | 0.243 | 1 | -2.288 | 0.043 | 1 |
| HEL-GB-A | 4.161 | 0 | 0 | 3.028 | 0.001 | 0.027 | 3.392 | 0 | 0.004 | 3.893 | 0 | 0.01 | 1.89 | 0.047 | 0.941 |
| BA1 | 1.248 | 0.029 | 1 | 0.14 | 0.841 | 1 | 0.538 | 0.395 | 1 | 1.377 | 0.025 | 1 | 0.736 | 0.251 | 1 |
| B79-G9 | 1.153 | 0.512 | 1 | -0.535 | 0.621 | 1 | 0.542 | 0.636 | 1 | 1.422 | 0.28 | 1 | -0.865 | 0.553 | 1 |
| Methanomethylophilaceae | 2.489 | 0 | 0.002 | -0.376 | 0.578 | 1 | -1.255 | 0.111 | 1 | -2.21 | 0.008 | 0.279 | -2.45 | 0.001 | 0.046 |
| **summer bacteria** | | | | | | | | | | | | | | | |
| Caldilineaceae | 0.532 | 0.624 | 1 | 0.409 | 0.448 | 1 | 0.065 | 0.923 | 1 | -0.99 | 0.473 | 1 | 0.637 | 0.339 | 1 |
| VadinHA17 | -0.299 | 0.58 | 1 | -0.534 | 0.124 | 1 | 0.045 | 0.918 | 1 | 0.032 | 0.949 | 1 | -0.693 | 0.248 | 1 |
| unidentified | 0.003 | 0.997 | 1 | -0.514 | 0.26 | 1 | 1.203 | 0.025 | 0.823 | -0.009 | 0.987 | 1 | -0.159 | 0.786 | 1 |
| Chromatiaceae | 0.288 | 0.556 | 1 | -0.215 | 0.59 | 1 | -0.395 | 0.49 | 1 | -0.895 | 0.303 | 1 | -1.132 | 0.225 | 1 |
| Desulfatiglandaceae | -0.62 | 0.162 | 1 | -0.721 | 0.133 | 1 | -0.066 | 0.889 | 1 | -1.029 | 0.028 | 0.994 | -1.873 | 0 | 0.009 |
| UBA11574 | -0.976 | 0.047 | 1 | -0.895 | 0.044 | 1 | -0.209 | 0.663 | 1 | -1.135 | 0.03 | 1 | -1.93 | 0.002 | 0.088 |
| Desulfocapsaceae | 0.717 | 0.053 | 1 | 0.082 | 0.833 | 1 | -0.346 | 0.507 | 1 | -0.151 | 0.82 | 1 | -0.746 | 0.502 | 1 |
| Flavobacteriaceae | -0.457 | 0.496 | 1 | -0.571 | 0.326 | 1 | -0.948 | 0.039 | 1 | -2.193 | 0 | 0.016 | -2.741 | 0.002 | 0.066 |
| UBA4823 | 0.175 | 0.703 | 1 | 0.38 | 0.469 | 1 | 1.078 | 0.04 | 1 | -0.222 | 0.751 | 1 | -0.74 | 0.328 | 1 |
| Anaerolineaceae | 0.201 | 0.688 | 1 | -0.383 | 0.436 | 1 | -0.191 | 0.665 | 1 | -0.843 | 0.044 | 1 | -1.228 | 0.024 | 1 |
| Thiobacillaceae | -0.978 | 0.052 | 1 | -1.351 | 0.013 | 0.417 | -1.504 | 0.039 | 1 | -2.364 | 0.006 | 0.224 | -3.25 | 0.001 | 0.052 |
| 34-128 | -0.88 | 0.218 | 1 | -0.644 | 0.219 | 1 | 1.768 | 0.04 | 0.923 | 0.713 | 0.316 | 1 | 0.38 | 0.595 | 1 |
| Cyanobiaceae | 0.052 | 0.91 | 1 | -0.098 | 0.815 | 1 | -0.397 | 0.381 | 1 | -1.289 | 0.163 | 1 | -2.192 | 0.015 | 0.581 |
| SZUA-229 | -0.815 | 0.45 | 1 | -1.031 | 0.065 | 1 | -0.746 | 0.247 | 1 | -2.172 | 0.035 | 1 | -2.71 | 0.001 | 0.052 |
| Ilumatobacteraceae | -0.613 | 0.674 | 1 | -0.896 | 0.171 | 1 | -1.155 | 0.105 | 1 | -0.693 | 0.322 | 1 | -0.501 | 0.462 | 1 |
| **winter bacteria** | | | | | | | | | | | | | | | |
| Caldilineaceae | 0.053 | 0.824 | 1 | -0.065 | 0.82 | 1 | -0.163 | 0.678 | 1 | -1.264 | 0.011 | 0.279 | -0.221 | 0.657 | 1 |
| VadinHA17 | -0.563 | 0.019 | 0.479 | -1.462 | 0 | 0 | -0.641 | 0.064 | 1 | -1.191 | 0.008 | 0.228 | -0.579 | 0.097 | 1 |
| Chromatiaceae | 0.271 | 0.325 | 1 | -0.112 | 0.688 | 1 | -0.254 | 0.434 | 1 | -1.232 | 0.041 | 1 | -0.393 | 0.424 | 1 |
| Thiobacillaceae | -0.545 | 0.064 | 0.986 | -0.914 | 0.01 | 0.27 | -1.27 | 0.006 | 0.187 | -3.014 | 0 | 0.012 | -2.286 | 0.001 | 0.038 |
| UBA11574 | -0.94 | 0 | 0.003 | -1.572 | 0 | 0 | -0.693 | 0.023 | 0.421 | -2.043 | 0 | 0.002 | -1.687 | 0.004 | 0.104 |
| Desulfatiglandaceae | -0.285 | 0.193 | 1 | -1.165 | 0 | 0.011 | 0 | 0.999 | 1 | -1.146 | 0.003 | 0.099 | -1.043 | 0.024 | 0.562 |
| JAAYZQ01 | 0.494 | 0.069 | 1 | -0.003 | 0.992 | 1 | 0.493 | 0.128 | 1 | -0.657 | 0.093 | 1 | -0.24 | 0.644 | 1 |
| Flavobacteriaceae | -1.868 | 0 | 0.002 | -2.03 | 0 | 0.007 | -1.547 | 0.001 | 0.028 | -3.259 | 0 | 0 | -3.432 | 0 | 0.001 |
| Desulfocapsaceae | -0.122 | 0.749 | 1 | -0.509 | 0.185 | 1 | -0.578 | 0.196 | 1 | -0.806 | 0.313 | 1 | -0.449 | 0.548 | 1 |
| EnvOPS12 | -0.271 | 0.23 | 1 | -1.038 | 0 | 0.018 | -0.704 | 0.062 | 1 | -1.872 | 0 | 0.004 | -1.426 | 0.022 | 0.559 |
| 34-128 | -1.147 | 0.001 | 0.022 | -1.517 | 0 | 0.001 | 1.438 | 0.002 | 0.026 | 0.553 | 0.336 | 1 | 0.632 | 0.165 | 1 |
| UBA4823 | 0.407 | 0.042 | 1 | -0.142 | 0.595 | 1 | 0.504 | 0.076 | 1 | -0.536 | 0.175 | 1 | -0.18 | 0.688 | 1 |
| Cyanobiaceae | 0.121 | 0.68 | 1 | -0.047 | 0.881 | 1 | -0.343 | 0.387 | 1 | -1.283 | 0.048 | 1 | -0.197 | 0.777 | 1 |
| Anaerolineaceae | -0.323 | 0.113 | 1 | -0.976 | 0 | 0.018 | -0.888 | 0.008 | 0.229 | -1.547 | 0 | 0.018 | -0.921 | 0.026 | 0.598 |

**Supplemental Figure S1** Temperature profile in the two bays between 2017 until 2023 as measured 1 m beneath the surface with the sampling points in this study marked by arrows. Grey shading shows the standard deviation (*n*=3) for the respective bays. Bottom water in the heated (⚫) and control (◼) bays are marked at the sampling times.


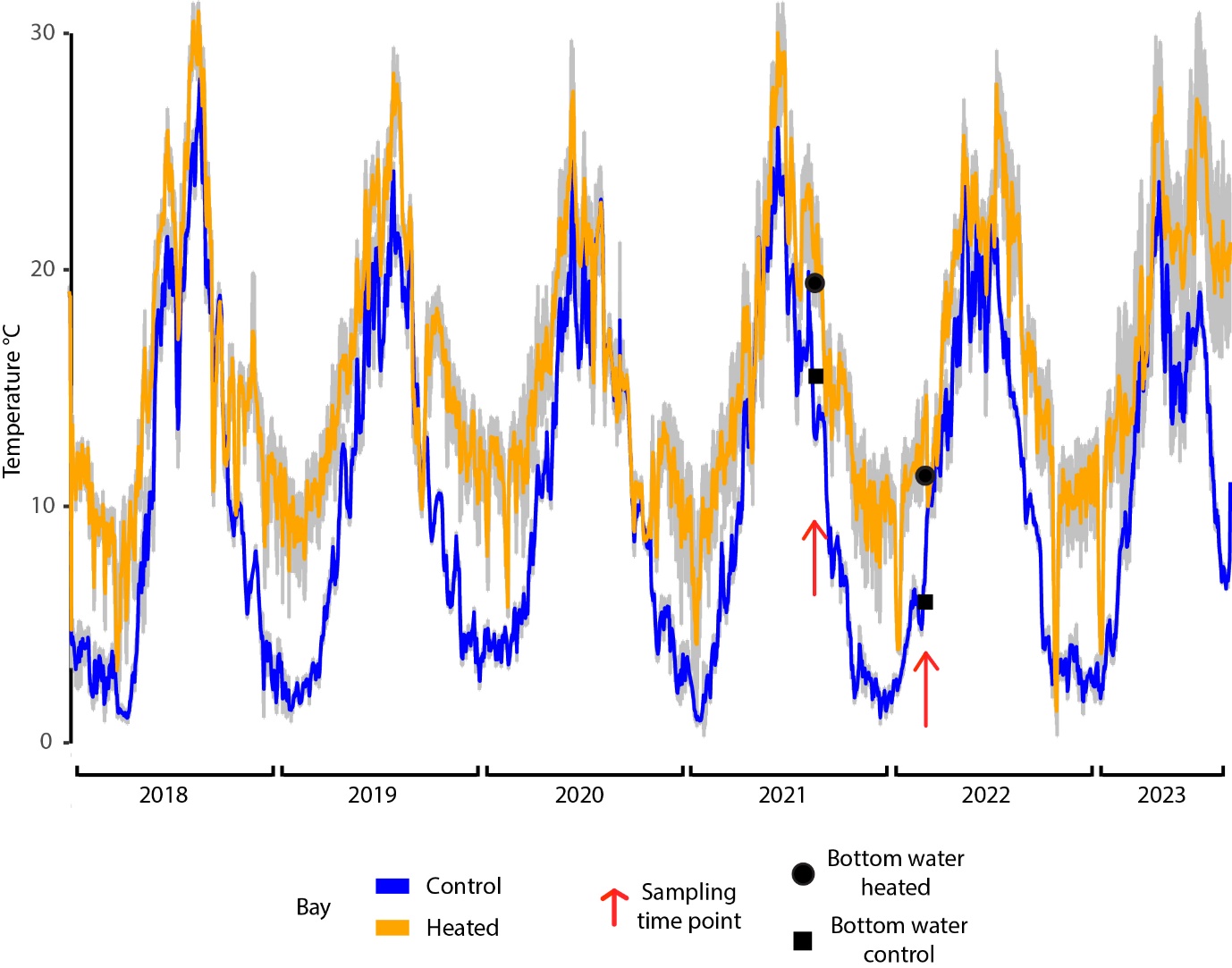


**Supplemental Figure S2** Sediment sample design showing duplicate cores taken at each sampling site and occasion with the sampling slices plus analysis plan for the various parameters.


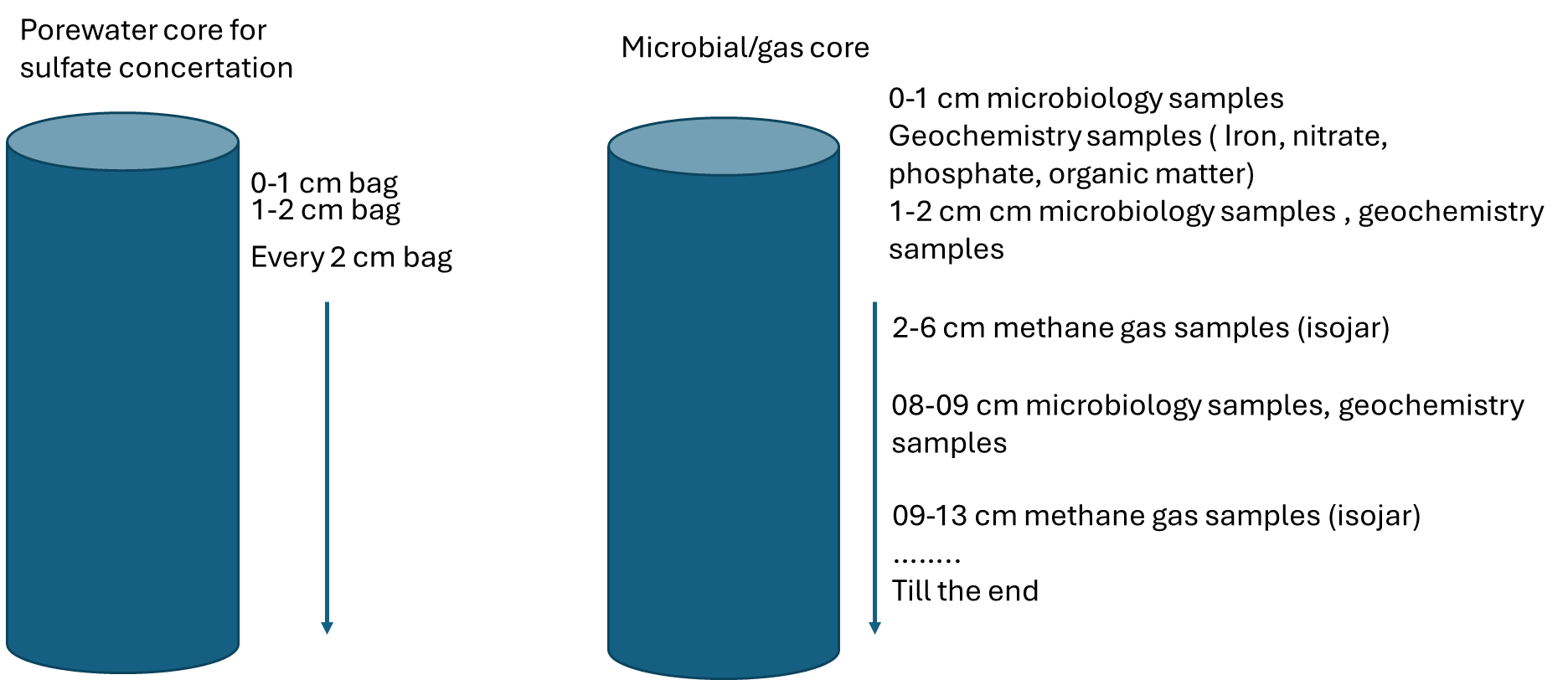


**Supplemental Figure S3** Rarefaction curves for all sequencing plates showing the number of amplicon sequence variants (ASVs) versus sequencing depth of 16S rRNA gene reads.

**Winter archaea sequencing**
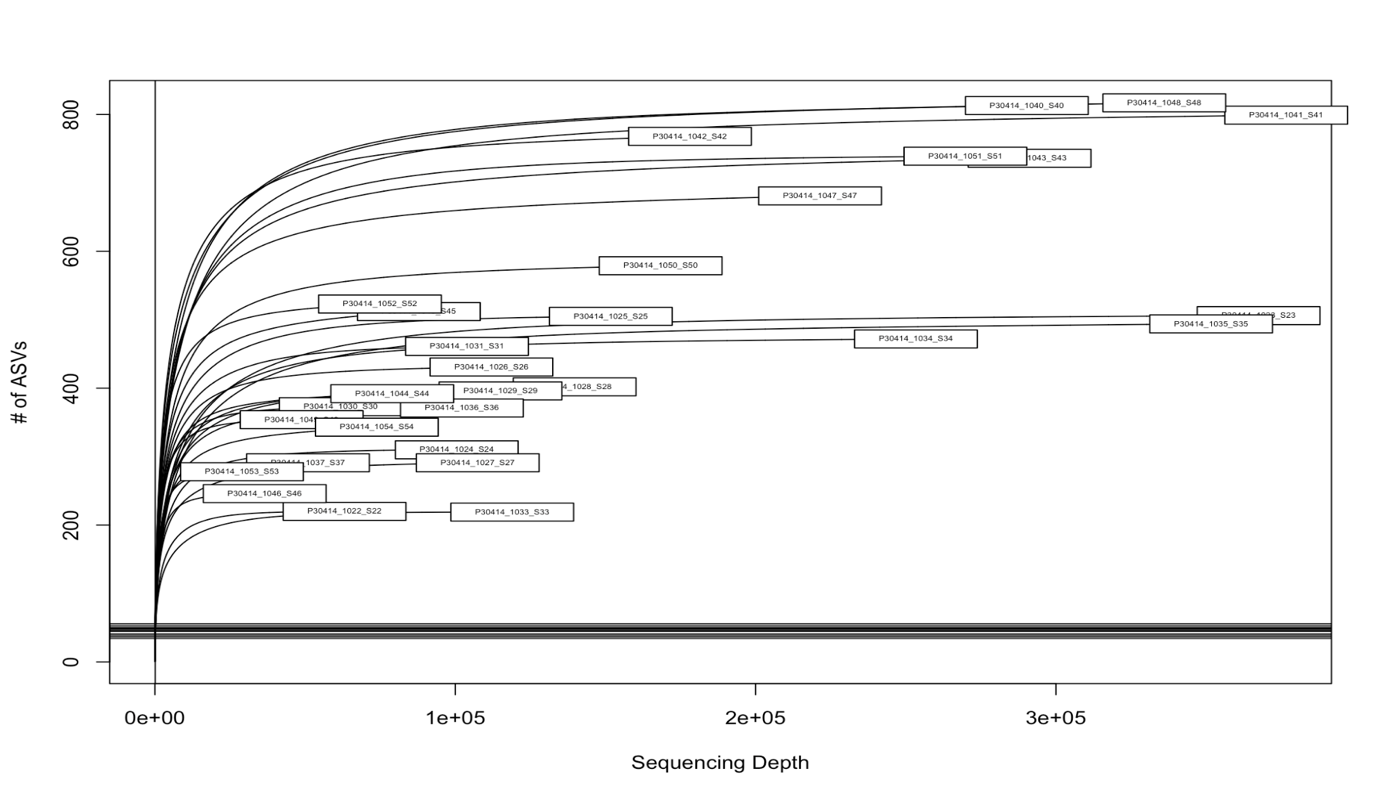


**Winter bacteria sequencing**


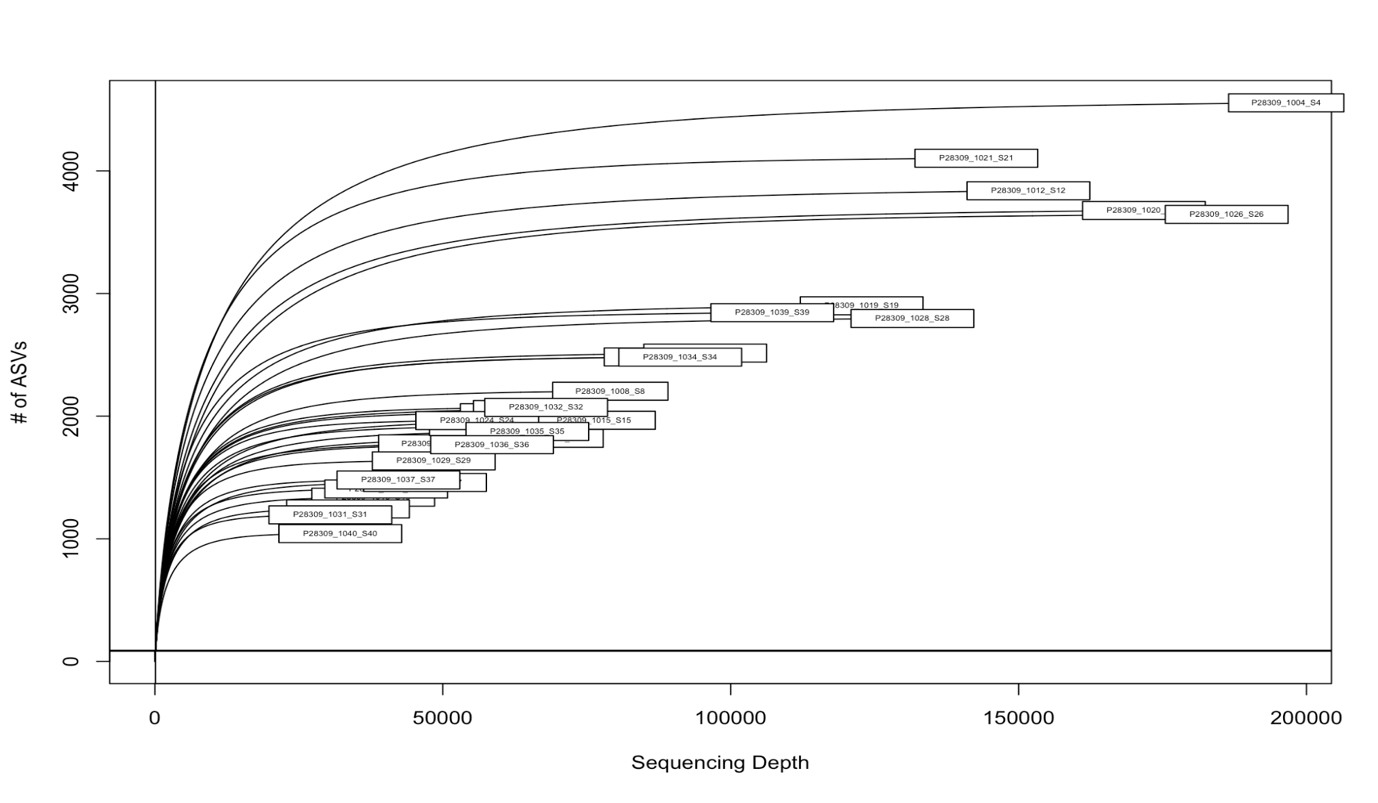


**Summer archaea sequencing**


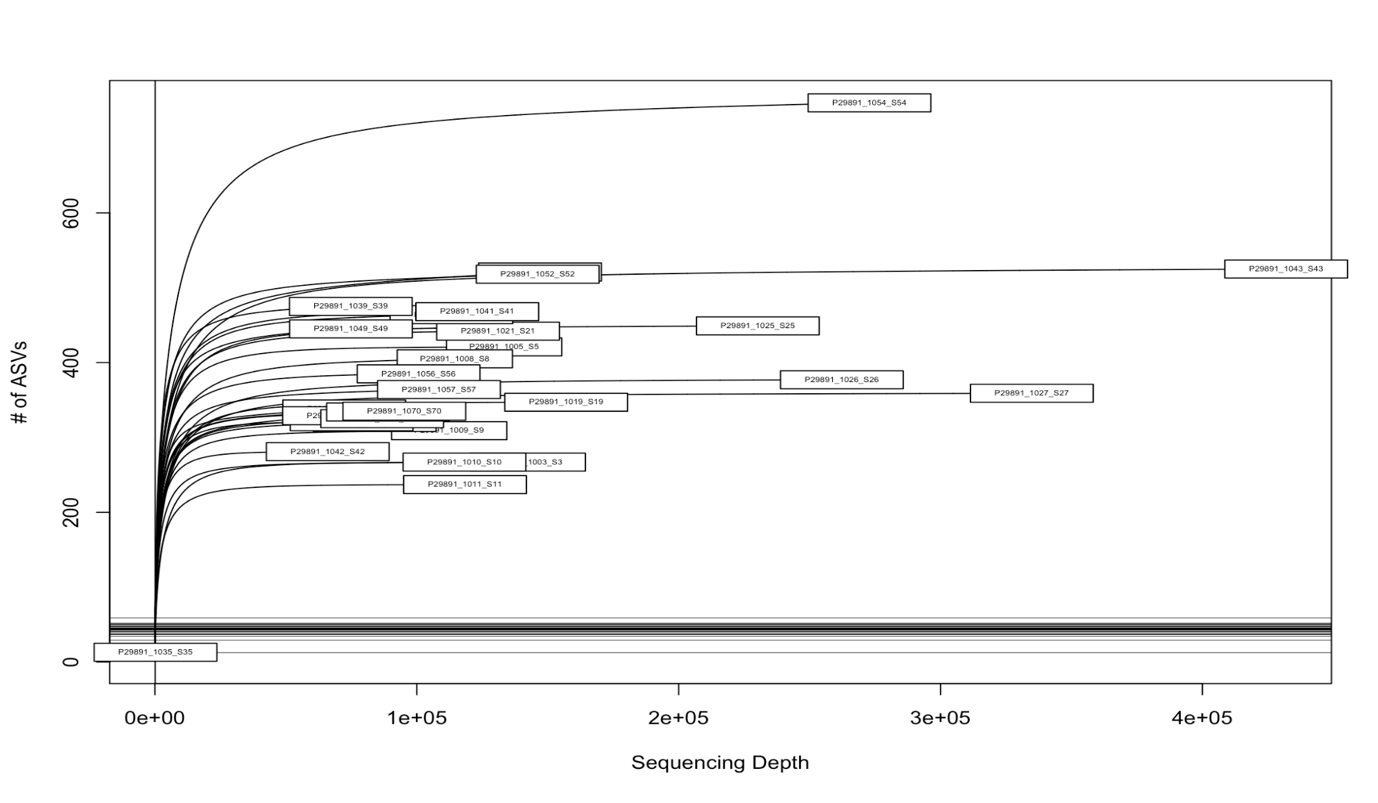


**Summer bacteria sequencing**


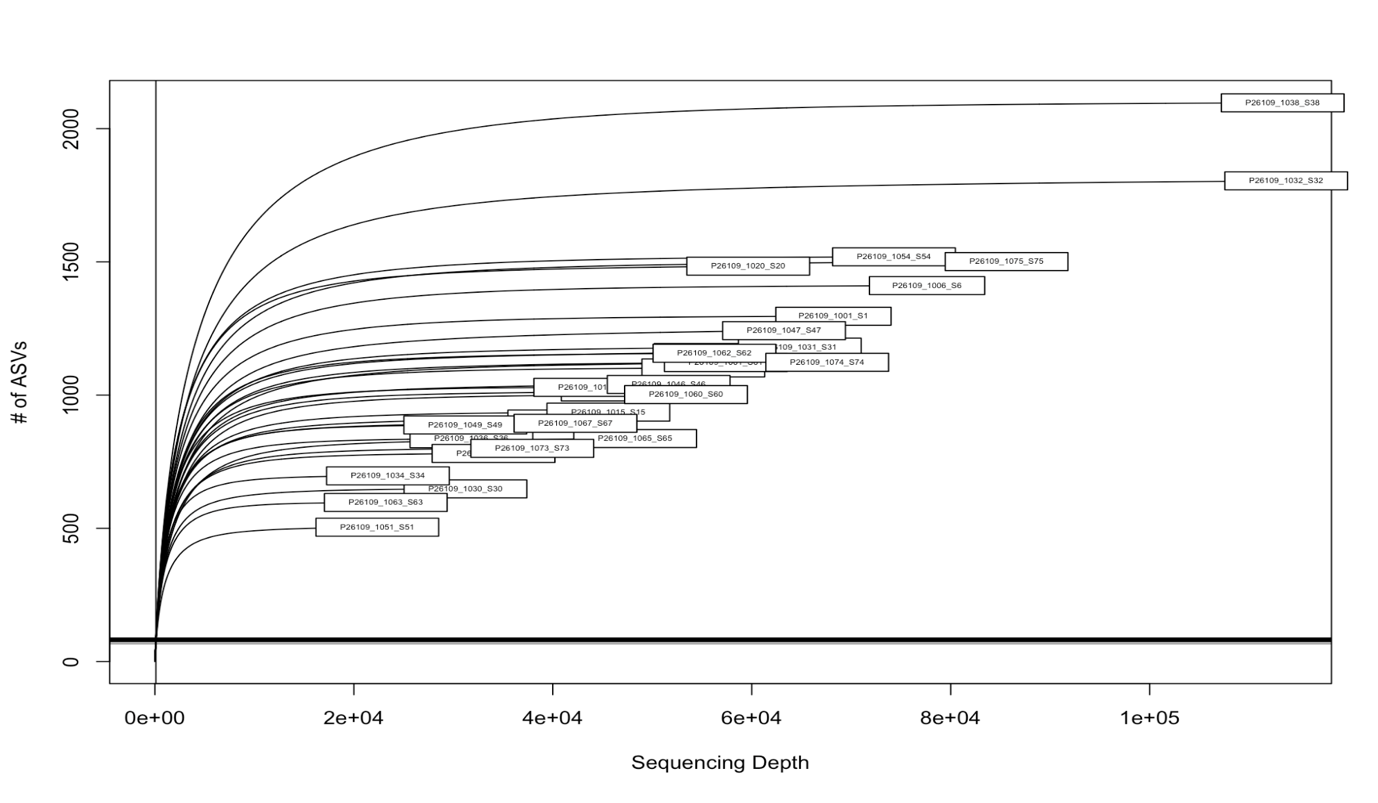


**Supplemental Figure S4** 16S rRNA gene-based relative abundances of archaeal and bacterial ASVs in each bay and depth during winter and summer at the levels of phylum, class, order, and genus.

**Phylum level**

**Class level**

**Order level**

**Genus level**

**Supplemental Figure S5** Redundancy analysis of the archaea (**A**) and bacteria (**B**) populations with geochemical parameters (including methane). Due to the sampling regime, methane concentrations were measured at different depths to the other samples and therefore, methane values from the closest corresponding depths were selected for representation.
